# Assessing Specialized Metabolite Diversity in the Cosmopolitan Plant Genus *Euphorbia* L.

**DOI:** 10.3389/fpls.2019.00846

**Published:** 2019-07-02

**Authors:** Madeleine Ernst, Louis-Félix Nothias, Justin J. J. van der Hooft, Ricardo R. Silva, C. Haris Saslis-Lagoudakis, Olwen M. Grace, Karen Martinez-Swatson, Gustavo Hassemer, Luís A. Funez, Henrik T. Simonsen, Marnix H. Medema, Dan Staerk, Niclas Nilsson, Paola Lovato, Pieter C. Dorrestein, Nina Rønsted

**Affiliations:** ^1^Natural History Museum of Denmark, Faculty of Science, University of Copenhagen, Copenhagen, Denmark; ^2^Collaborative Mass Spectrometry Innovation Center, Skaggs School of Pharmacy and Pharmaceutical Sciences, University of California, San Diego, La Jolla, CA, United States; ^3^Skaggs School of Pharmacy and Pharmaceutical Sciences, University of California, San Diego, La Jolla, CA, United States; ^4^Bioinformatics Group, Department of Plant Sciences, Wageningen University & Research, Wageningen, Netherlands; ^5^Comparative Plant and Fungal Biology, Royal Botanic Gardens, Richmond, United Kingdom; ^6^Department of Botany, Federal University of Santa Catarina, Florianópolis, Brazil; ^7^Department of Biotechnology and Biomedicine, Technical University of Denmark, Lyngby, Denmark; ^8^Department of Drug Design and Pharmacology, Faculty of Health and Medical Sciences, University of Copenhagen, Copenhagen, Denmark; ^9^Front End Innovation, LEO Pharma A/S, Ballerup, Denmark; ^10^Center for Microbiome Innovation, University of California, San Diego, La Jolla, CA, United States

**Keywords:** computational metabolomics, coevolution, *Euphorbia*, specialized metabolites, immunomodulatory testing

## Abstract

Coevolutionary theory suggests that an arms race between plants and herbivores yields increased plant specialized metabolite diversity and the geographic mosaic theory of coevolution predicts that coevolutionary interactions vary across geographic scales. Consequently, plant specialized metabolite diversity is expected to be highest in coevolutionary hotspots, geographic regions, which exhibit strong reciprocal selection on the interacting species. Despite being well-established theoretical frameworks, technical limitations have precluded rigorous hypothesis testing. Here we aim at understanding how geographic separation over evolutionary time may have impacted chemical differentiation in the cosmopolitan plant genus *Euphorbia*. We use a combination of state-of-the-art computational mass spectral metabolomics tools together with cell-based high-throughput immunomodulatory testing. Our results show significant differences in specialized metabolite diversity across geographically separated phylogenetic clades. Chemical structural diversity of the highly toxic *Euphorbia* diterpenoids is significantly reduced in species native to the Americas, compared to Afro-Eurasia. The localization of these compounds to young stems and roots suggest a possible ecological relevance in herbivory defense. This is further supported by reduced immunomodulatory activity in the American subclade as well as herbivore distribution patterns. We conclude that computational mass spectrometric metabolomics coupled with relevant ecological data provide a strong tool for exploring plant specialized metabolite diversity in a chemo-evolutionary framework.

## Introduction

Likely as a consequence of niche colonization and adaptive evolution, plants produce an extraordinary variety of molecules. These so-called specialized metabolites are deployed in the response to a multitude of biotic as well as abiotic factors (Bednarek and Osbourn, 2009), for example, to attract pollinators or seed-dispersing animals, in signaling between other plants and symbiotic microorganisms or to combat herbivores or physical stresses such as UV light (Bednarek and Osbourn, 2009; Wink, 2010).

Coevolutionary theory suggests that an arms race between plants and herbivores is the major driving force yielding increased specialized metabolite diversity in plants (Ehrlich and Raven, 1964; Becerra, 2007; Richards et al., 2015). The evolution of a chemically different and biologically more active molecule increases a plant’s fitness by reducing the fitness of its herbivores (Ehrlich and Raven, 1964; Firn and Jones, 2003), and the probability of producing one or more biologically active compounds may increase with plant specialized metabolite diversity (Jones and Firn, 1991). Previous studies have assessed plant specialized metabolite diversity in relation to herbivory and, in agreement with the coevolutionary theory, found a positive relationship between plant chemical diversity, the degree of insect herbivore trophic specialization and diversity (Becerra, 2007; Richards et al., 2015). Furthermore, the geographic mosaic theory of coevolution predicts that coevolutionary interactions vary across geographic scales. Patterns of reciprocal response in coevolving species differ across populations. This variation may be caused by fluctuating biotic and abiotic factors across landscapes, causing species to coevolve in some populations but not in others (Thompson, 2005a,b). For example, detoxification profiles of the parsnip webworm *Depressaria pastinacella* (Duponchel, 1838) and plant defense profiles of its host, the wild parsnip (*Pastinaca sativa* L.) were significantly mismatched in the presence of an alternate host plant, the cow parsnip (*Heracleum lanatum* Michx.). This suggests that the presence of a chemically different alternate host can affect selection intensity and patterns of reciprocal response across landscapes (Zangerl and Berenbaum, 2003). Traits, which are shaped by coevolving interactions become only fixed in a species when strong positive natural selection is present across all populations (Thompson, 2005b). Alternatively, particularly in widespread species, an inevitable consequence of the geographic mosaic of coevolution is the formation of highly divergent populations that have the potential to form new species (Thompson, 2008).

If we assume that the main driving force of plant specialized metabolite diversity is a coevolutionary arms race, and coevolutionary interaction is a rare event and constrained by a geographic mosaic of species interactions, we would expect significant differences in specialized metabolite diversity in plant phylogenetic clades, which have evolved in geographically very distant regions. This would be because we expect geographically very distant regions to vary most in biotic as well as abiotic factors influencing coevolutionary species interaction. While a strong reciprocal selection on the interacting plant–herbivore species might promote plant chemical diversity in one region, the same combination of biotic factors is not likely to be found in another significantly distant geographic region. For example, this could be due to the absence of one of the interacting species.

Previous studies have assessed the role of plant–herbivore interactions in the evolution of plant specialized metabolite diversity (Becerra, 2007; Richards et al., 2015); however, they have not explicitly taken geography into account. Here, we investigate the role of biogeography in the evolution of specialized metabolite diversity in 43 *Euphorbia* species, representing the genus’ global genetic diversity and biogeographic history across all continents. To do so, we acquired untargeted mass spectrometry data including mass fragmentation (MS/MS) data to assess the specialized metabolome. To annotate and compare the specialized metabolite content across all 43 species, we use a combination of mass spectral molecular networking, *in silico* annotation tools, substructure recognition, and automated chemical classification leading to the semi-automated annotation of over 1800 specialized metabolites, up to the level of chemical structures, substructures, or subclasses. Furthermore, we assess the possible ecological relevance of plant specialized metabolite diversity through cell-based high-resolution immunomodulatory bioactivity profiling, 3D mass spectral molecular cartography, and herbivore distribution patterns.

**FIGURE 1:**
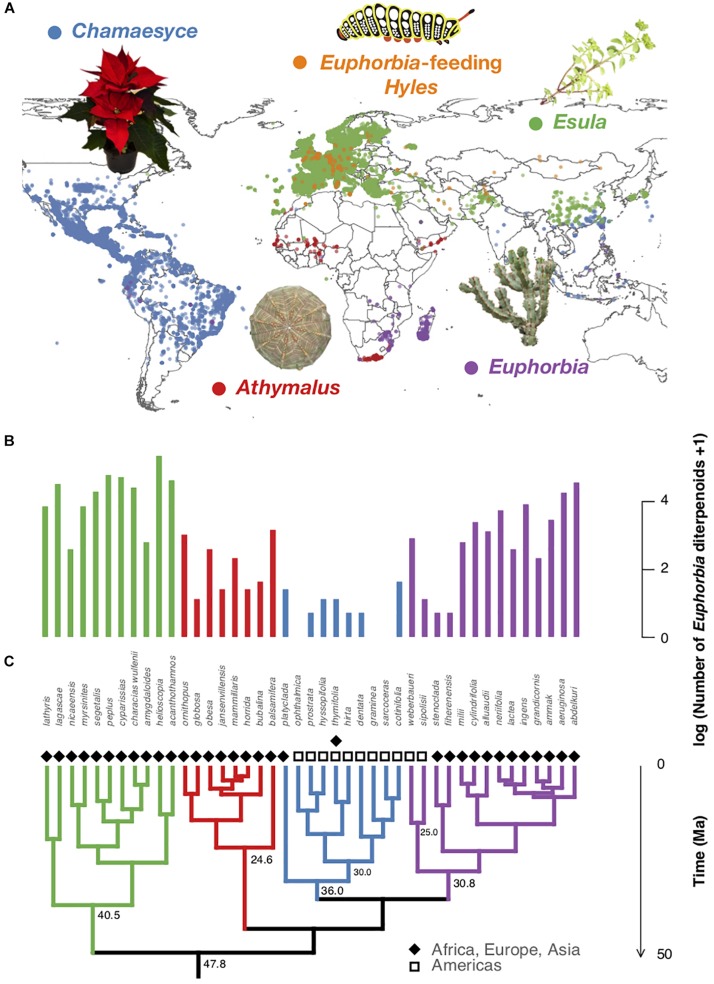
Biogeography, phylogenetic relationships, and diterpenoid production of representative *Euphorbia* species. **(A)** Occurrences of *Euphorbia* species investigated chemically and *Euphorbia*-feeding *Hyles* moth larvae retrieved from GBIF and manually restricted to native areas. **(B)** Number of putatively annotated *Euphorbia* diterpenoids per species analyzed. **(C)** *Euphorbia* phylogenetic tree (50% majority rule consensus tree from Bayesian analysis of 11,587 bp of DNA markers spanning all three plant genomes: chloroplast, mitochondrial, nuclear). Species of subg. *Esula* exhibit a high number of diterpenoids and co-occur with larvae of *Euphorbia*-feeding *Hyles*, whereas the American radiation of subg. *Chamaesyce* shows reduced *Euphorbia* diterpenoid production. Subgeneric clades are highlighted with different colors: *Athymalus* (red), *Chamaesyce* (blue), *Esula* (green), and *Euphorbia* (purple). Photos: Mogens Trolle and Madeleine Ernst.

**FIGURE 2:**
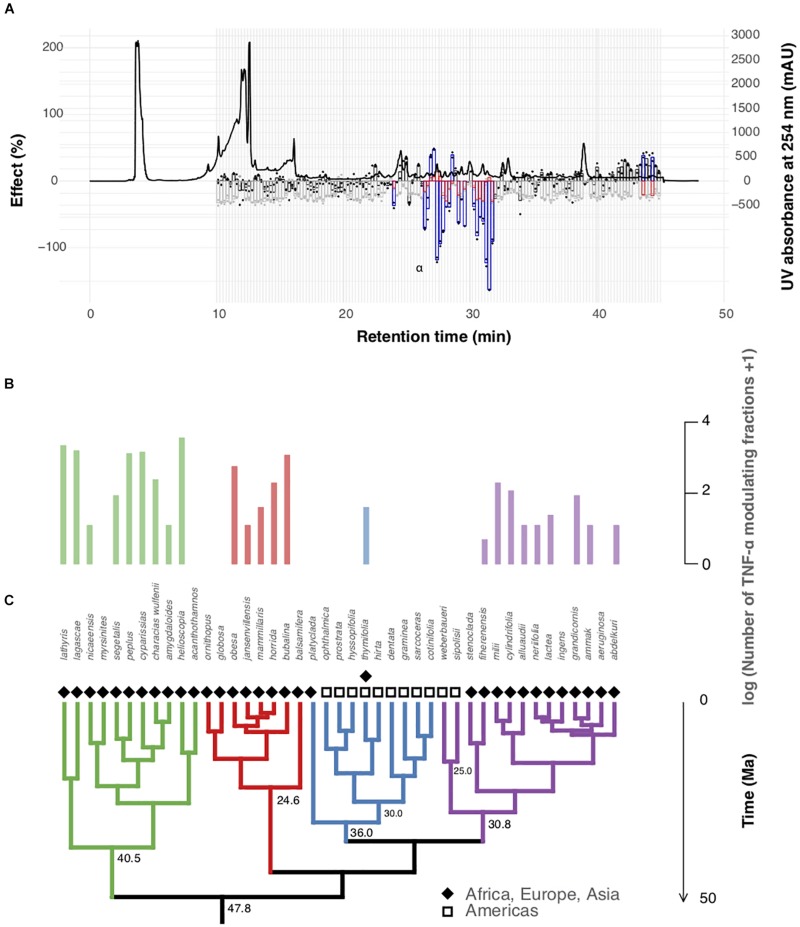
Pro-inflammatory potential of representative *Euphorbia* species. **(A)** TNF-α modulating properties of fractions of the crude extract of *Euphorbia peplus*. The effect of the fractionated extract on TNF-α modulation (black/blue) and cell viability (gray/red) is plotted against the HPLC chromatogram at 254 nm. Fractions with significant modulation of TNF-α are highlighted in blue, whereas fractions significantly modulating cell viability are highlighted in red. 100% effect corresponds to 100% inhibition of TNF-α release and cell viability, respectively. **(B)** Number of fractions significantly modulating TNF-α without having a significant effect on cell viability per species analyzed. **(C)** *Euphorbia* phylogenetic tree (50% majority rule consensus tree from Bayesian analysis of 11,587 bp of DNA markers spanning all three plant genomes: chloroplast, mitochondrial, nuclear). Species of subg. *Esula* exhibit a high number of TNF-α modulating fractions and co-occur with larvae of *Euphorbia*-feeding *Hyles* (Figure 1), whereas the American radiation of subg. *Chamaesyce* shows reduced TNF-α modulating activity. Subgeneric clades are highlighted with different colors: *Athymalus* (red), *Chamaesyce* (blue), *Esula* (green), and *Euphorbia* (purple).

*Euphorbia* is among the most diverse and species-rich plant genera on Earth, exhibiting a near-cosmopolitan distribution and extraordinary chemical diversity among 2,000 species (Horn et al., 2012, 2014; Vasas and Hohmann, 2014). Most recent phylogenetic studies have supported four geographically strongly separated subgeneric clades (Horn et al., 2012). The genus is estimated to have originated in Africa approximately 48 million years ago and expanded to the American continents through two single long-distance dispersal events 30 and 25 million years ago (Figures 1, 2; Zimmermann et al., 2010; Yang and Berry, 2011; Horn et al., 2012, 2014; Yang et al., 2012; Dorsey et al., 2013; Peirson et al., 2013; Riina et al., 2013). The subgenera *Athymalus* (Neck.) Rchb. and *Esula* Pers. exhibit a primarily Afro-Eurasian radiation, subg. *Euphorbia* is diverse in its distribution range and subg. *Chamaesyce* Raf. comprises the majority of the American radiation of the genus (Horn et al., 2012). The genus’ chemical diversity is characterized by an extraordinary diversity of macro- and polycyclic diterpenoids, biosynthetically derived from a head-to-tail cyclization of the tetraprenyl pyrophosphate precursor (Shi et al., 2008; Vasas and Hohmann, 2014; Appendino, 2016). These compounds are known to play an important ecological role as feeding deterrents and have shown exclusive occurrence and chemotaxonomic relevance in the plant families Euphorbiaceae and Thymelaeaceae (Batra, 1983; Hundsdoerfer et al., 2005a; Huang et al., 2014; Vasas and Hohmann, 2014; Hua et al., 2017). *In vitro Euphorbia* diterpenoids are known to induce different immunomodulatory responses through the selective modulation of protein kinase C (PKC) including the release of prostaglandins and pro-inflammatory cytokines such as TNF-α, interleukin-6, and interleukin-1β (Smith and Meldrum, 1992; Berman, 2012; Uto et al., 2012; Jian et al., 2018; Zhang et al., 2019).

Following coevolutionary and geographic mosaic theory, we hypothesize that (1) there are significant differences in specialized metabolite diversity across *Euphorbia* phylogenetic clades, which is (2) correlated with geographical separation through evolutionary time with highest differences observed between phylogenetic clades having evolved in the American versus the Afro-Eurasian continents, where genetic interchange is least likely, and (3) specialized metabolite diversity in *Euphorbia* may reflect a role in herbivory defense, as predicted by herbivore–plant coevolutionary hotspots.

## Materials and Methods

### Collection of Plant Material

Forty-three *Euphorbia* species (Supplementary Data S1) were collected from the greenhouses of the Living Collections of the Botanical Garden, Natural History Museum of Denmark, University of Copenhagen. Live plants were sampled for xerophytic species, whereas herbaceous perennials were grown from seeds originating from the seed bank of the Botanical Garden or collections performed in southern Brazil (species 11–14 and 16–18). *Euphorbia myrsinites* L. and *E. amygdaloides* L. were purchased as live plants from Jespers Planteskole, Holstebro A/S, Denmark and Kridtvejs Planter, Denmark, respectively, and kept in the greenhouse of the Botanical Garden with the other herbaceous species until harvest. To obtain representative samples of the specialized metabolite profile in the living plants, the plants were sampled as whole intact specimens with the roots included. Over six specimens or as many as available from the collections were sampled for each species (Supplementary Data S1). Some tree species or rare species could not be sampled as whole plants, in these cases, branches of representative parts of the living specimen were sampled (Supplementary Data S1). The fresh plant material was then stored at −20°C until extract preparation. Voucher specimens were prepared for species with enough material available from the Living Collections and deposited at Herbarium C, Natural History Museum of Denmark, Copenhagen. Taxonomic species identity was confirmed for all species sampled based on the voucher specimens, the living specimens in the greenhouse or photographs.

### Collection of Plant Material for 3D Mass Spectral Molecular Cartography

Individual plant parts from two to three specimens of one representative species of each subgeneric clade of *Euphorbia* were sampled (*E. horrida* Boiss., subg. *Athymalus*, three specimens; *E. hirta* L., subg. *Chamaesyce*, two specimens; *E. lathyris* L., subg. *Esula*, two specimens; *E. milii* Des Moul., subg. *Euphorbia*, two specimens). Approximately 200 mg fresh plant material of each individual plant part was collected in 1.5 ml Eppendorf tubes and flash frozen under liquid nitrogen. The samples were stored at −80°C until further analysis.

### Extract Preparation

Individual specimens of the same species were pooled, and the frozen plant material was disrupted with a pestle and mortar in liquid nitrogen and approximately 75 g was extracted with 975 ml ethyl acetate (VWR Chemicals, HiPerSolv Chromanorm) under sonication (Fisher Scientific) at 40°C during 2 h. The extracts were filtered and evaporated to dryness on a rotary evaporator. The dried extracts were then resuspended in acetonitrile (VWR Chemicals, HiPerSolv Chromanorm), extracted under sonication (Fisher Scientific) at 40°C during 15 min, filtered, and evaporated to dryness. Description of the extract preparation for 3D mass spectral molecular cartography is provided in the Supplementary Methods.

### LC–MS/MS Analysis

Extracts were transferred to a 96-well plate (Falcon, 96-well plates, 0.34 ml, polypropylene) and dried with a vacuum centrifuge. Samples were redissolved in 3/7 vol/vol methanol (Fisher Scientific, HPLC Grade)/acetonitrile (Fisher Scientific, Optima LC/MS) to a concentration of 10 mg/ml, in a volume of 150 μl, with a 100 mM concentration of ammonium formate, sealed with Zone-Free Sealing Film (Excel Scientific), and centrifuged for 30 min at 2,000 r.p.m. at 4°C. Ammonium formate was used to promote the ionization of diterpene esters as ammonium adducts rather than sodiated adducts, thus inducing a richer MS/MS fragmentation pattern as described by Vogg et al. (1999). MS analysis was performed on a qTOF Maxis II (Bruker Daltonics) mass spectrometer with an electrospray ionization (ESI) source, controlled by OTOF control and Hystar. Additional descriptions of the liquid chromatography tandem mass spectrometry (LC–MS/MS) analysis as well as a description of the LC–MS/MS analysis for 3D mass spectral molecular cartography is provided in the Supplementary Methods.

### High-Resolution TNF-α Modulation Profiling

*Euphorbia* diterpenoids are known to induce different immunomodulatory responses *in vitro* through the selective modulation of PKC including the release of prostaglandins and pro-inflammatory cytokines such as TNF-α, interleukin-6, and interleukin-1β (Smith and Meldrum, 1992; Berman, 2012; Uto et al., 2012; Jian et al., 2018; Zhang et al., 2019). Both vertebrates and invertebrates produce cytokines, and it is likely that they exhibit similar functions both in insect as well as mammalian cells (Adamo, 2008). To assess the immunomodulatory capacity of compounds or compound groups in *Euphorbia*, we chose to measure *in vitro* TNF-α release from peripheral blood mononuclear cells (PBMCs). TNF-α was chosen above other cytokines because of its key role in initiating local inflammatory responses (Monaco et al., 2014; Sedger and McDermott, 2014; Kalliolias and Ivashkiv, 2016). First, we subjected the same 43 plant extracts we had previously characterized chemically to high-resolution microplate-based extract collection, resulting in 144 individual fractions per extract. Then we used all the individual fractions to treat human peripheral blood mononuclear cells (hPBMCs), which are activated to release TNF-α by stimulation with anti-CD3- and anti-CD28-coated beads. Finally, we assessed the modulation of TNF-α by evaluating the level of TNF-α released in culturing media in the HPLC trace (Wubshet et al., 2013; Supplementary Data S2 and Supplementary Figures S2–S45). Detailed description of the high-resolution microplate-based extract collection as well as the TNF-α inhibition assay is provided in the Supplementary Methods.

### Mass Spectral Molecular Networking

Liquid chromatography tandem mass spectrometry data of the pooled extracts were converted to mzML data, and lock mass correction was performed using Compass Data Analysis (Bruker Daltonics). MassFuser merged mzML files (for a detailed description, see Supplementary Methods) were then processed using Optimus v1.1.0, a processing workflow for LC–MS/MS untargeted metabolomics based on OpenMS algorithms (Röst et al., 2016; Protsyuk et al., 2018)^1^ with parameters set to: MS polarity mode positive, *m/z* tolerance 30 ppm, noise threshold 20,000, half of MS/MS isolation window: 0.02 Da, RT tolerance of MS/MS acquisition 5 s, RT tolerance 20 s, enable re-integration of missing features off, enable pose clustering alignment off, intensity factor compared to blanks 3, minimal occurrence number 1, presence of MS/MS on, variation in pooled QC runs and replicates off, enable feature normalization off, save as MGF, MS1 noise threshold 250, and MS2 noise threshold 150. Features detected in blank samples were discarded from the samples using the Optimus Workflow. Subsequently, the ms2.mgf output file was uploaded to the Global Natural Products Social (GNPS) Molecular Networking web server^2^ and submitted to network analysis using the following settings: Precursor ion mass tolerance 0.02 Da, fragment ion mass tolerance 0.02 Da, min pairs Cos 0.5, min matched fragment ions 6, Network TopK 10, minimum cluster size 1, maximum connected component size 200, and Run MS Cluster off. The molecular networks were visualized with Cytoscape version 3.4.0 (Shannon et al., 2003). The data are publicly accessible at http://gnps.ucsd.edu under the MassIVE accession no. MSV000081082 and network exploration options and views are available and networking parameters described at: https://gnps.ucsd.edu/ProteoSAFe/status.jsp?task=26326c233918419f8dc80e8af984cdae. Description of the mass spectral molecular networking analysis for 3D mass spectral molecular cartography is provided in the Supplementary Methods.

### Integration of *in silico* Annotation, Automated Chemical Classification, and Substructure Recognition With Mass Spectral Molecular Networks

*In silico* structure annotation was performed by submitting the preprocessed ms1-2.mgf output file of the pooled extracts from Optimus to Sirius+CSI:FingerID (Dührkop et al., 2015; Böcker and Dührkop, 2016) with *m/z* tolerance set to 20 ppm. Additionally, data were submitted to Network Annotation Propagation (NAP) (da Silva et al., 2018). For NAP, both [M+NH4]+ and [M+H]+ adducts were searched with *m/z* tolerance set to 15 ppm and parameters described at: https://proteomics.ucsd.edu/ProteoSAFe/status.jsp?task=184a80db74334668ae1d0c0f852cb77c and https://proteomics2.ucsd.edu/ProteoSAFe/status.jsp?task=2cfddd3b8b1e469181a13e7d3a867a6f.

We matched a custom database of molecular structures against our samples’ preprocessed mass spectral data using both Sirius+CSI:FingerID and NAP. This database was compiled manually from literature (Shi et al., 2008; Vasas and Hohmann, 2014) and the dictionary of natural products (DNP)^3^. Subsequently, *in silico* structure matches from Sirius+CSI:FingerID and NAP were submitted to automated chemical classification using ClassyFire^4^ (Djoumbou Feunang et al., 2016) and consensus classifications at each hierarchical level of the chemical taxonomy per mass spectral molecular subnetwork were calculated. Consensus classifications and molecular structures were then visualized on the molecular networks using Cytoscape version 3.4.0 (Shannon et al., 2003). Substructure recognition of the crude extracts was performed by submitting the preprocessed ms2.mgf output file from Optimus to MS2LDA (van der Hooft et al., 2016; Wandy et al., 2018). Data and parameters used are publically accessible at http://ms2lda.org/basicviz/short_summary/390/. Subsequently substructures (Mass2Motifs) were mapped on the nodes of the mass spectral molecular network, and Mass2Motifs shared among different nodes were mapped on the edges connecting the nodes and visualized using Cytoscape version 3.4.0. Description of the integration of *in silico* annotation, automated chemical classification, and substructure recognition with mass spectral molecular networks for 3D mass spectral molecular cartography is provided in the Supplementary Methods.

### 3D Modeling and Visualization

We converted approximately 250 photos taken from one to three specimens of one representative species of each subgeneric clade of *Euphorbia* into high-definition 3D meshes using Autodesk Remake^5^. The 3D meshes were then exported to .obj format and edited using Meshmixer^6^. Point coordinates for sampled plant parts were added using Meshlab (Cignoni et al., 2008), and the 3D Models were exported to .stl format. Representative samples were taken of individual plant parts (e.g., roots, upper and lower leaves and stems, fruits), rather than exact sample locations (e.g., three to five representative pieces of one inflorescence were taken and pooled together for LC–MS/MS analysis, the entire inflorescence was subsequently mapped with the pooled LC–MS data). The 3D mass spectral mapping thus shows data obtained from representative pools of individual plant parts, rather than being accurate point representatives of plant parts sampled. Point coordinates and LC–MS data were combined into a .csv file and were then mapped on the 3D Models using ’ili^7^ (Bouslimani et al., 2015; Floros et al., 2017). URL links of cartographical snapshots, which can be opened in a web browser can be found in Supplementary URL S1, Links 1–15. Each URL will open the ’ili web application, and load data, settings, and visualizations as shown in Figure 5 and Supplementary Figures S3–S9. Additionally, the visualization is interactive, and the models can be rotated, visualization parameters changed, and other molecules selected by their retention time and *m/z* value (Protsyuk et al., 2018).

### Calculation of the Chemical Structural Compositional Similarity and Chemogram

To assess specialized metabolite diversity in relation to the evolutionary history of *Euphorbia*, we calculated the chemical structural and compositional similarity (CSCS) per *Euphorbia* subgeneric clade (Sedio et al., 2017). CSCS provides a more accurate estimate of phytochemical diversity compared to traditional dissimilarity measures (e.g., Bray-Curtis) by integrating the cosine scores retrieved from the mass spectral molecular network analysis and thus also accounting for chemical structural similarity among specialized metabolites detected by mass spectrometry (Sedio, 2017; Sedio et al., 2017). To illustrate chemical similarity among species we created a chemogram by using the pairwise chemical structural and compositional dissimilarities (CSCDs), corresponding to 1-CSCS, as input for a hierarchical cluster analysis with the complete agglomeration method.

### Phylogenetic Hypothesis and Comparative Methods

We produced a phylogenetic hypothesis of *Euphorbia* compiling DNA sequences from 10 markers spanning all three plant genomes (cp accD, cp rbcL-accD, cp ndhF, cp rbcL, cp rpl16, cp trnL-F, mt nad1B-C, mt rps3, nu EMB2765, nu ITS) of a publicly available dataset (Horn et al., 2012, 2014). Our matrix included sequences (11,587 base pairs) of 38 out of the 43 *Euphorbia* species investigated chemically. We produced a Bayesian phylogenetic hypothesis using parameters described in Horn et al. (2014). Subsequently, we added missing species (*E. sarcoceras* O.L.M. Silva and Cordeiro, *E. hyssopifolia* L., *E. ophthalmica* Pers., *E. prostrata* Aiton, *E. thymifolia* L.) based on their subgeneric classification (Yang et al., 2012; da Silva and Cordeiro, 2016) using the bind.tip function of the R package phytools (Revell, 2012) to the 50% majority rule consensus tree. We performed a phylogenetic generalized least squares (PGLS) regression analysis using the pgls function of the R package caper (Orme et al., 2013) to test whether the number of putatively annotated molecules within a chemical subclass and the number of TNF-α modulating fractions are significantly associated over evolutionary time. PGLS is a modification of the generalized least squares regression, taking into account non-independence of species sharing an evolutionary history by weighing the generalized least squares regression by the amount of expected correlation between species based on their phylogenetic relationships (Symonds and Blomberg, 2014). We only assessed chemical subclasses containing at least five molecules and non-zero values for at least 20 species out of the 43 species within the phylogenetic tree. Supplementary Table S1 shows chemical subclasses and associated *p*-values. Figure 1 and Supplementary Figure S12 show the number of TNF-α modulating fractions versus the number of putatively annotated molecules within the chemical subclasses with significant *p*-values (*Euphorbia* diterpenoids, all diterpenoids, glycosylglycerols) across the phylogenetic tree. The best fit was observed for the *Euphorbia* diterpenoids (Figure 1), with species of the American clade within subg. *Chamaesyce* being almost entirely deprived of these compounds, corresponding to the TNF-α modulating properties of these species.

### Species Occurrences of Sampled *Euphorbia* and *Euphorbia*-Feeding *Hyles*

We downloaded species occurrences of sampled *Euphorbia* species and *Euphorbia*-feeding *Hyles* from the Global Biodiversity Information Facility (GBIF^8^). Before mapping species occurrences, we reduced the datasets to the native distributions of the respective species (Meerman, 1993; Hundsdoerfer et al., 2005b; Govaerts et al., 2014). Species names of *Euphorbia*-feeding *Hyles* were retrieved from Hundsdoerfer et al. (2009). URL links to GBIF species occurrence downloads are found below:

Species of subg. *Euphorbia*: GBIF.org (5 January 2018) GBIF Occurrence Download https://doi.org/10.15468/dl.llmwkb.

Species of subg. *Esula*: GBIF.org (5 January 2018) GBIF Occurrence Download https://doi.org/10.15468/dl.avy89i.

Species of subg. *Chamaesyce*: GBIF.org (5 January 2018) GBIF Occurrence Download https://doi.org/10.15468/dl.9ntc6n.

Species of subg. *Athymalus*: GBIF.org (5 January 2018) GBIF Occurrence Download https://doi.org/10.15468/dl.ug6cnm.

*Hyles euphorbiae*: GBIF.org (5 January 2018) GBIF Occurrence Download https://doi.org/10.15468/dl.cgwykr.

Other *Euphorbia*-feeding *Hyles* species: GBIF.org (24 January 2018) GBIF Occurrence Download https://doi.org/10.15468/dl.ahwr3w.

### Code and Data Availability

All scripts used for data analysis were written in R version 3.3.2^9^. Data, code, and material required to understand and assess the conclusions of this research are publicly available at https://github.com/DorresteinLaboratory/supplementary-GlobalEuphorbiaStudy. LC–MS/MS data are publicly accessible on GNPS under the MassIVE accession nos. MSV000081082 and MSV000081083. Bayesian trees used for analysis were deposited on dryad^10^.

## Results

**FIGURE 3:**
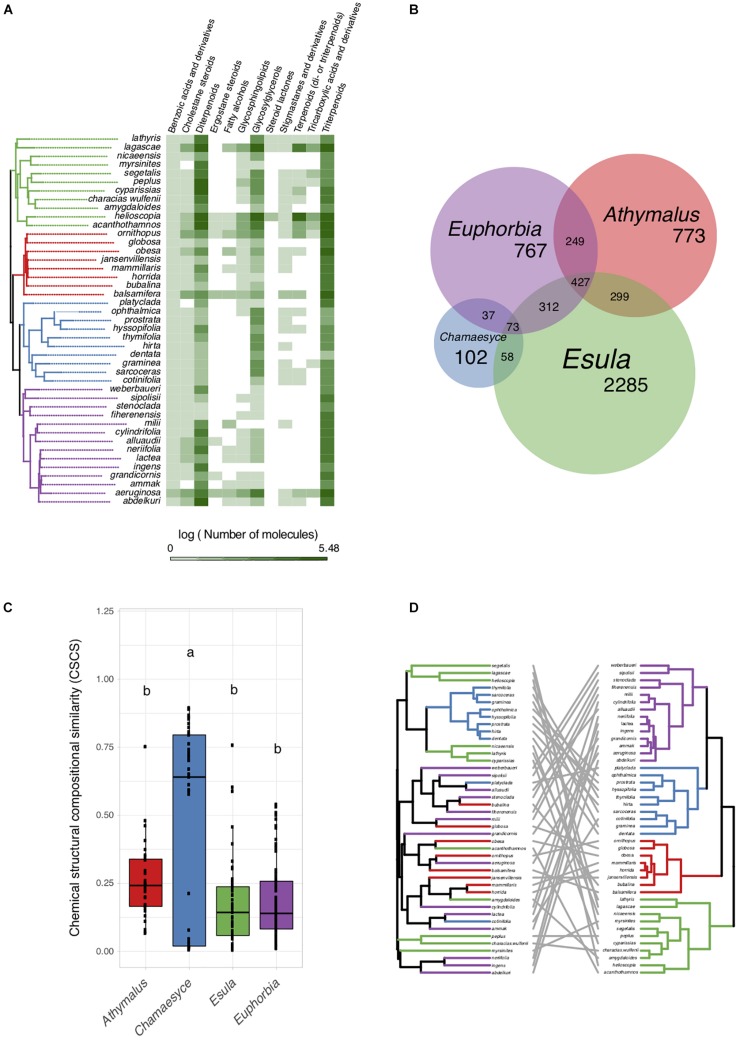
Specialized metabolite diversity in *Euphorbia*. **(A)** Distribution of specialized metabolite classes on the *Euphorbia* phylogenetic tree (50% majority rule consensus tree from Bayesian analysis of 11,587 bp of DNA markers spanning all three plant genomes: chloroplast, mitochondrial, nuclear). Chemical classes of *Euphorbia* specialized metabolites were putatively identified using a mass spectrometry based workflow combining mass spectral molecular networking, *in silico* annotation, automated chemical classification, and substructure recognition. **(B)** Molecular features representing individual mass spectral molecular network nodes shared across species of *Euphorbia* subgeneric clades. **(C)** Chemical similarity among *Euphorbia* subgeneric clades assessed using the chemical structural compositional similarity. Compared to subgenera *Athymalus*, *Esula*, and *Euphorbia*, subg. *Chamaesyce* exhibits very few chemically distinct features and high chemical structural compositional similarity. **(D)** *Euphorbia* chemogram (left) and phylogenetic tree (right). The chemogram was generated using hierarchical cluster analysis on the pair-wise chemical structural and compositional dissimilarities of the tandem mass spectrometry data of the crude extracts using the complete agglomeration method. Phylogeny and chemogram show low overlap, suggesting that closely related *Euphorbia* species differ considerably in their chemistry.

To assess specialized metabolite diversity in relation to the evolutionary and biogeographic history of *Euphorbia*, we subjected extracts of 43 *Euphorbia* species to LC–MS/MS, created mass spectral molecular networks through GNPS Molecular Networking (Watrous et al., 2012; Wang et al., 2016), and calculated the CSCS for all *Euphorbia* subgeneric clades (Sedio et al., 2017). Our data show significantly higher chemical similarity among species of the predominantly American subg. *Chamaesyce* compared to the mean chemical similarity among species of the remaining subgeneric clades mostly native to Afro-Eurasia (Figure 3). The only species clustered in the chemogram (sharing high chemical similarity) are eight out of nine species representing the American radiation within subg. *Chamaesyce* (Figure 3D).

**FIGURE 4:**
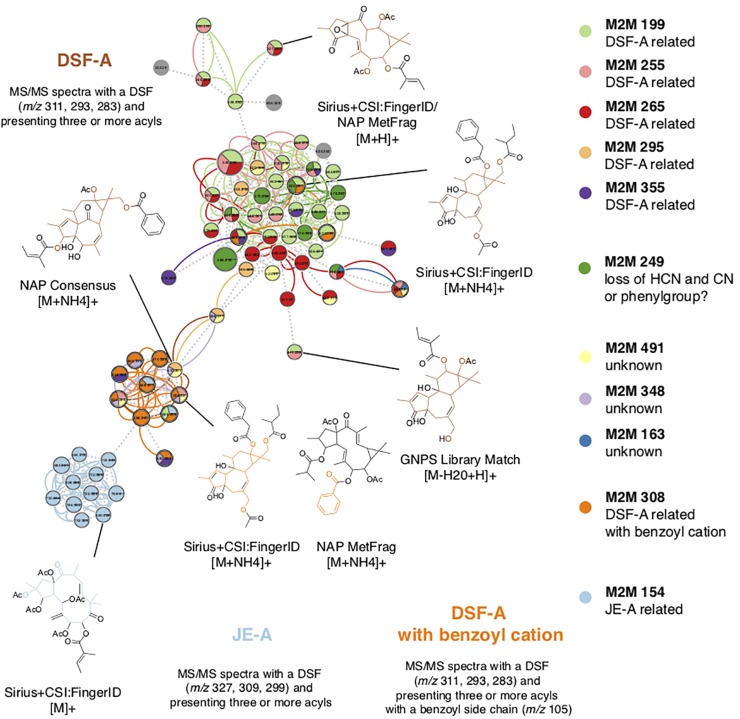
Putative identification of chemical compound classes. We putatively identified compound classes within the mass spectral molecular networks by combining *in silico* annotation with automated chemical classification and substructure recognition (MS2LDA). *Euphorbia* diterpenoids exhibit many isoforms; therefore, different diterpene backbone skeletons were found within the same molecular family. Matching substructures (Mass2Motifs) associated with diterpenoid substructures obtained from matches to reference spectra and *in silico* structure annotation enabled the identification of different diterpene spectral fingerprints clustered within one molecular family. Node size represents the total ion current (TIC) of all samples analyzed, edge colors represent different substructures (Mass2Motifs) that are shared across different nodes, and dotted lines connecting the nodes represent the cosine score. M2M, Mass2Motif; DSF-A, diterpene spectral fingerprint type A; JE-A, Jatrophane ester type A.

To further understand the chemo-evolutionary relationships of *Euphorbia* at a molecular level we putatively identified major specialized metabolite classes by combining mass spectral molecular networking with *in silico* annotation tools (Dührkop et al., 2015; Böcker and Dührkop, 2016; da Silva et al., 2018), substructure recognition (van der Hooft et al., 2016; Wandy et al., 2018), and automated chemical classification through ClassyFire (Djoumbou Feunang et al., 2016). This allowed us to putatively identify chemical classes within the mass spectral molecular networks, as well as chemical subclasses within molecular families (Figure 4 and Supplementary Figure S2). Automated chemical classification revealed annotated compound classes for over 30% of the compounds detected, corresponding to a level 3 metabolite identification according to the Metabolomics Standard Initiative’s reporting standards (Sumner et al., 2007; Supplementary Figures S1, S2 and Supplementary Notes S1). The genus *Euphorbia* is known for producing an extraordinary diversity of macro- and polycyclic diterpenoids, biosynthetically derived from a head-to-tail cyclization of the tetraprenyl pyrophosphate precursor (Shi et al., 2008; Vasas and Hohmann, 2014). To distinguish these diterpenoids from diterpenoids biosynthetically derived from a cascade cyclization, we here refer to them as *Euphorbia* diterpenoids. Many known *Euphorbia* diterpenoids as well as other metabolite classes were revealed. Out of six major structural classes of *Euphorbia* specialized metabolites (sesquiterpenoids, diterpenoids, cerebrosides, phenolics, flavonoids, and triterpenoids including steroids) (Shi et al., 2008; Vasas and Hohmann, 2014), four are found in our mass spectral molecular networks (i.e., diterpenoids; triterpenoids including cholestane and ergostane steroids; steroid lactones and stigmastanes; and glycosylglycerols corresponding to cerebrosides). Additionally, *in silico* structure annotation suggests the presence of tricarboxylic and benzoic acids and derivatives as well as fatty alcohols and glycosphingolipids (Figure 3A). Among the *Euphorbia* diterpenoids, we observe different skeletal types within the same molecular families [two or more connected components of a graph) (Figure 4)]. Many *Euphorbia* diterpenoid backbone skeletons are isomeric, and their respective fragmentation spectra are highly similar (Nothias-Scaglia et al., 2015). Nonetheless, we were able to distinguish different diterpene spectral fingerprints within a molecular family by mapping Mass2Motifs on the mass spectral molecular networks. Mass2Motifs correspond to common patterns of mass fragments and neutral losses, which are extracted using unsupervised substructure discovery of the MS/MS data through MS2LDA (van der Hooft et al., 2016; Wandy et al., 2018). Consistent with the low chemical diversity exhibited by subg. *Chamaesyce* and previous observations of *Euphorbia* diterpenoids exhibiting anti-herbivore biological activity (Batra, 1983; Hundsdoerfer et al., 2005a; Huang et al., 2014; Hua et al., 2017), we find very few to no *Euphorbia* diterpenoids in representatives of the American radiation of subg. *Chamaesyce* (Figure 1).

**FIGURE 5:**
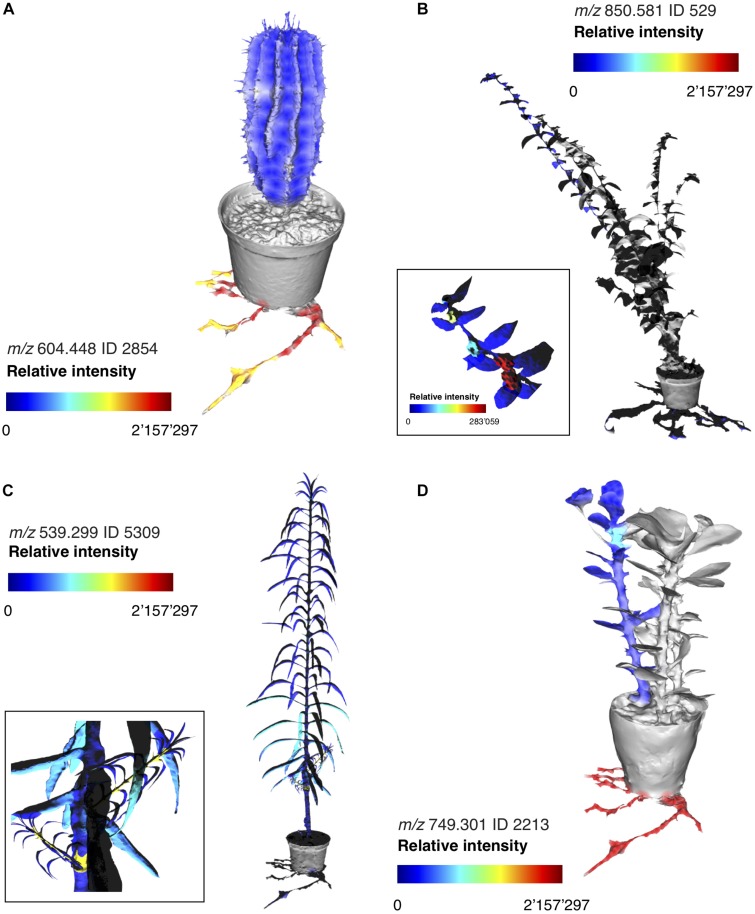
Molecular maps of selected *Euphorbia* diterpenoids across representatives of each subgeneric clade. Relative intensity of LC–MS molecular features annotated as *Euphorbia* diterpenoids through spectral matching **(A)** *Euphorbia horrida*, subg. *Athymalus*, **(B)** *Euphorbia hirta*, subg. *Chamaesyce*, **(C)** *Euphorbia lathyris*, subg. *Esula*, and **(D)** *Euphorbia milii* var. *hislopii*, subg. *Euphorbia*. For interactive cartographical snapshots, see Supplementary URL S1, Links 1–15. The 3D images are for illustrative purposes only and do not represent exact locations of sample collection.

Assessing intra-specimen distribution of specialized metabolites can reveal possible ecological functions. Specialized metabolites allocated for the defense against herbivory, for example, can be highly compartmentalized and enriched only in specialized structures such as secretory ducts or laticifers found in roots or stems, as a strategy used by the plants to avoid autotoxicity (Engelberth, 2015). Furthermore, young plant parts may be enriched in defense molecules as a consequence of higher nutritional quality and increased rate of herbivory (Coley and Aide, 1991; Coley and Barone, 1996; Brenes-Arguedas et al., 2006; Kursar et al., 2009). To understand where the *Euphorbia* diterpenoids are produced within the plants, we dissected four species representing the four subgeneric clades into approximately 20 sections (Supplementary Figures S10, S11). Mass spectrometric investigation revealed that diterpenoids are primarily found in the roots, in representatives of subgenera *Euphorbia* and *Athymalus E. milii* var. *hislopii* (N.E.Br.) Ursch & Leandri and *E. horrida*; Figure 5 and Supplementary Figures S3, S6–S9). In the European subg. *Esula* (*E. lathyris*), diterpenoid production is also pronounced in other plant parts, such as the young stems (Figure 4 and Supplementary Figure S5). Consistent with the lower chemical diversity reported above, diterpenoid production is reduced or absent from most sections throughout the whole plant in *E. hirta*, a representative of the American clade within subg. *Chamaesyce* (Figure 5 and Supplementary Figures S4, S10). Compartmentalization of the diterpenoids to mainly young stems and roots could suggest possible functions as anti-feeding molecules (McKey, 1979; Batra, 1983; Hundsdoerfer et al., 2005a; Huang et al., 2014; Hua et al., 2017).

As anti-herbivore activity cannot be directly tested from the past continental transition and data on *Euphorbia* herbivory at a global scale are very scarce, we set out to test a bioactivity that could reflect a chemical defense mechanism. One strategy of defense invoked by plants to overcome their sessile habit is through local immunomodulatory and inflammatory effects on herbivores. Previous studies (Huang et al., 2014; Hua et al., 2017) have reported anti-feeding activity of selected *Euphorbia* diterpenoids against generalist plant-feeding insects, *Spodoptera exigua* (Hübner, 1808) and *Helicoverpa armigera* (Hübner, 1808). *In vitro*, *Euphorbia* diterpenoids are known to induce different immunomodulatory responses through the selective modulation of PKC including the release of prostaglandins and pro-inflammatory cytokines such as TNF-α, interleukin-6, and interleukin-1β (Smith and Meldrum, 1992; Berman, 2012; Uto et al., 2012; Jian et al., 2018; Zhang et al., 2019). Here, we evaluated the pro-inflammatory potential of the crude *Euphorbia* extracts by measuring the capacity of small extract fractions, corresponding to compounds or compound groups in each of the 43 *Euphorbia* species that modulate *in vitro* TNF-α release from PBMCs. Both vertebrates and invertebrates produce cytokines, and assuming they may exhibit similar functions both in insect as well as in mammalian cells (Adamo, 2008), this assay setup may be considered an approximation of potential anti-herbivore activity, independent of the species, as *Euphorbia* may experience attack by both vertebrates and invertebrates (Batra, 1983; Loutit et al., 1987; Hundsdoerfer et al., 2005a; Huang et al., 2014; Hua et al., 2017). Figure 2 and Supplementary Figures S2–S45 in Supplementary Data S2 show TNF-α modulating properties of all 43 *Euphorbia* fractionated extracts, where fractions with significant modulation of TNF-α without having a significant effect on cell viability (Supplementary Methods S8) are highlighted in blue. Several fractions with significant modulation of TNF-α are found in representatives of subgenera *Athymalus*, *Esula*, and *Euphorbia* (Supplementary Figures S2–S9, S20–S45 in Supplementary Data S2), whereas only one out of nine representatives (*Euphorbia thymifolia*; Supplementary Figure S19 in Supplementary Data S2) of the American subg. *Chamaesyce* is found to contain immunomodulatory compounds or compound groups. To address the role of *Euphorbia* diterpenoids involved with TNF-α modulating properties, we tested for correlation between the number of TNF-α modulating fractions and the number of molecules within the previously annotated chemical classes using PGLS regression analysis. The association between the number of TNF-α modulating fractions and the number of *Euphorbia* diterpenoids was found significant (*p*-value: 0.02) (Figures 1B,C, Supplementary Figure S12, and Supplementary Table S1), supporting our hypothesis of a possible ecological function of *Euphorbia* diterpenoids as immunomodulatory defense molecules.

Finally, to explore the possibility of *Euphorbia* diterpenoids being produced as a consequence of local plant–herbivore coevolutionary hotspots, we searched for known *Euphorbia* herbivores in the literature. Several hawk moth species of the genus *Hyles* were found to be highly specialized herbivores of *Euphorbia* (Batra, 1983; Hundsdoerfer et al., 2009). They have been shown to exhibit host specificity and to tolerate the highly toxic *Euphorbia* diterpenoids, which they reuse as a defense strategy against their own predators by regurgitating plant material from the gut (Batra, 1983; Hundsdoerfer et al., 2005a; Huang et al., 2014; Hua et al., 2017). Native species distribution data suggest a close co-occurrence of *Euphorbia*-feeding *Hyles* species with the chemically highly diverse and biologically active European species of subg. *Esula* (Figure 1A), and an absence in the American habitats of the chemically less diverse and biologically little active representatives of subg. *Chamaesyce*. This finding supports the hypothesis of co-evolutionary hotspots driving chemical defense diversity. However, further evidence linking specialized metabolite profiles with *Hyles* herbivory, tolerance mechanisms, or other possible mammalian or insect herbivory and distribution would be needed to draw a strong conclusion.

## Discussion

Following coevolutionary theory, we hypothesized that specialized metabolite diversity would show significant phylogenetic structure in *Euphorbia*, which could be driven by the chemical structural diversity of metabolites potentially used in the defense against herbivory. Furthermore, as a consequence of herbivore–plant coevolutionary hotspots, we would observe significant differences in specialized metabolite diversity across phylogenetic clades geographically separated through evolutionary time.

Our results show that specialized metabolite diversity as well as the number of *Euphorbia* diterpenoids with previously reported feeding-deterrent properties (Batra, 1983; Hundsdoerfer et al., 2005a; Huang et al., 2014; Hua et al., 2017) is significantly reduced in phylogenetic clades native to the Americas, compared to species native to the Afro-Eurasian continents. The localization of these compounds to young stems and roots as well as reduced immunomodulatory activity in species of the American subclade further support their possible ecological function as immunomodulatory defense molecules. Furthermore, these findings could suggest chemo-evolutionary adaptation to reduced herbivory pressure in the Americas. High specialized metabolite diversity, number of defensive molecules and immunomodulatory activity, on the other hand, suggests a coevolutionary arms race in species native to the Afro-Eurasian continents. *Euphorbia* originated in Africa and only later expanded to the Americas (Zimmermann et al., 2010; Yang and Berry, 2011; Horn et al., 2012, 2014; Yang et al., 2012; Dorsey et al., 2013; Peirson et al., 2013; Riina et al., 2013). Thus, if the maintenance of the chemical coevolutionary defensive traits come at a cost, species of the American subclade are indeed most likely to have lost this trait, as maintenance would require an equally specialized herbivore in the newly occupied habitats. Our results are consistent with this hypothesis with species of the American radiation of subg. *Chamaesyce* exhibiting least chemical structural diversity, none to very few *Euphorbia* diterpenoids as well as immunomodulatory activity.

If *Euphorbia* specialized metabolite diversity is a consequence of herbivore–plant coevolutionary hotspots, we would expect specialized *Euphorbia* herbivores to geographically co-occur with chemically highly diverse and biologically active *Euphorbia* species. Native species distribution data suggest a close co-occurrence of *Euphorbia*-feeding *Hyles* species with the chemically highly diverse and biologically active European species of subg. *Esula* (Figure 1A). On the other hand, *Euphorbia*-feeding *Hyles* are not native to the American habitats, where chemically less diverse and biologically little active representatives of subg. *Chamaesyce* occur (Ernst et al., 2018, Preprint). This supports the hypothesis that specialized metabolite diversity in *Euphorbia* may be a consequence of herbivore–plant coevolutionary hotspots. However, our data also suggest that African members of subg. *Athymalus* and subg. *Euphorbia* occurring in Southern Africa and Madagascar, outside of the distribution range of *Euphorbia*-feeding *Hyles*, produce a high diversity of feeding deterrent diterpenoids. We speculate that previously not described (or extinct) generalist or specialist *Euphorbia*-feeding herbivores occur (or occurred) in these regions, which contributed to maintaining adaptive pressure. The black rhinoceros, *Diceros bicornis* (Linnaeus, 1758) distributed in the southern African subregion, for example, was found to feed often and extensively on African *Euphorbia* species of subg. *Euphorbia* (Loutit et al., 1987). Anecdotal evidence of the lack of specialized herbivores in the Americas also corroborate with our results. A single hawk moth species, *Hyles euphorbiae*, was only introduced recently to North America (Batra, 1983), where it was used as a host-specific enemy and biological control for the reportedly highly invasive European species of subg. *Esula* (*E. cyparissias* L. and *E. esula* L.), lacking herbivores in the newly occupied habitats. Furthermore, although the poinsettia (*E. pulcherrima* Willd. ex Klotzsch), a very well-known ornamental plant and American representative of subg. *Chamaesyce*, is notorious for its “extreme toxicity” among the general public, toxicity remains unconfirmed in the clinic, as 92.4% of patients exposed to the plant did not develop adverse effects (Krenzelok et al., 1996). This corroborates with our results of the low chemical diversity, diterpenoid content, and immunomodulatory activity found in representatives of subg. *Chamaesyce*.

Research exploring the geographic variation of plant–herbivore co-evolution can increase our understanding of the processes that generate and maintain plant chemical diversity across landscapes (Dyer et al., 2018). Here we assessed the evolution of specialized metabolite diversity in relation to the biogeographic history of the cosmopolitan genus *Euphorbia*. Although extensive genetic studies would be necessary to reveal evidence for the coevolutionary relationship across *Euphorbia* species and its herbivores, our study reveals for the first time potential evolutionary drivers of specialized metabolite diversity at a global scale.

Plant specialized metabolite diversity is an important driver of many aspects of biodiversity, among others shaping trophic interactions, species diversity, and ecological coexistence (Richards et al., 2015; Sedio, 2017). The evolutionary origins and ecological consequences of specialized metabolite diversity have been addressed by several well-established hypotheses in chemical ecology; however, technical limitations in assessing chemical structures in high-throughput have so far precluded rigorous hypothesis testing (Bednarek and Osbourn, 2009; Richards et al., 2015; Allard et al., 2016; Sedio, 2017; Dyer et al., 2018). Consequently, most previous studies have either focused on broad chemical classes of compounds (Ayres et al., 1997; Agrawal et al., 2009; Kursar et al., 2009), a small number of well-characterized molecules (Ayres et al., 1997; Becerra, 1997, 2007; Kursar et al., 2009), or a large quantity of unidentified chemical entities (Richards et al., 2015; Sedio et al., 2017). Here we performed the semi-automated putative identification of over 30% of the metabolites detected up to the level of chemical structures or subclasses. This corresponds to more than five times as many identified metabolites when compared to most recent studies using mass spectral metabolomics techniques to investigate plant specialized metabolite diversity (Sedio et al., 2017). Compared to previous studies, our approach thus represents a significant technical advance and reveals an unprecedented insight into plant chemical diversity in *Euphorbia*. Computational mass spectrometric metabolomics tools introduced here are likely to revolutionize our understanding of evolutionary and ecological mechanisms underlying plant chemical diversity in the future.

## Code and Data Availability

The datasets generated for this study can be found in https://massive.ucsd.edu/, MSV000081082, and MSV000081083.

## Author Contributions

ME, L-FN, CS-L, OG, HS, NN, DS, PD, and NR designed the study. ME and KM-S collected and sampled *Euphorbia* species from the Botanical Garden at the Natural History Museum of Denmark. GH and L-AF collected seeds of Brazilian *Euphorbia* species and performed taxonomic circumscription and identification. ME prepared the plant extracts. L-FN performed LC-MS/MS analysis of the pooled Euphorbia extracts. ME performed LC-MS/MS analysis for the 3D mass spectral molecular cartography. ME and L-FN performed mass spectral molecular networking analysis. JvdH performed MS2LDA analysis. L-FN, JvdH, and ME annotated Mass2Motifs on MS2LDA. ME and JvdH developed and performed the (semi-)automated annotation workflow by combining mass spectral molecular networking with MS2LA, in silico annotation and ClassyFire. RS developed dereplication strategies through NAP and provided support for data analysis. NN, PL, DS, and ME designed the high-resolution bioactivity study. ME performed the high-resolution bioactivity study and analyzed data together with DS, NN, and PL. ME generated the phylogenetic hypothesis, performed comparative analyses and wrote all scripts used for data analysis. ME wrote the manuscript together with L-FN, NR, and PD. All authors discussed the results and commented on the manuscript.

## Conflict of Interest Statement

The authors declare that the research was conducted in the absence of any commercial or financial relationships that could be construed as a potential conflict of interest.

## Footnotes

1 https://github.com/MolecularCartography/Optimus

2 http://gnps.ucsd.edu

3 http://dnp.chemnetbase.com

4 http://classyfire.wishartlab.com/

5 https://remake.autodesk.com/

6 http://www.meshmixer.com/

7 https://github.com/MolecularCartography/ili

8 https://www.gbif.org

9 http://www.R-project.org/

10 https://doi.org/10.5061/dryad.t513409

## References

[B1] Adamo S. A. (2008). “6 - Bidirectional connections between the immune system and the nervous system in insects,” in *Insect Immunology*, ed. Beckage N. E. (San Diego, CA: Academic Press), 129–149. 10.1016/b978-012373976-6.50008-2

[B2] Agrawal A. A. Salminen J. P. Fishbein M. (2009). Phylogenetic trends in phenolic metabolism of milkweeds (Asclepias): evidence for escalation. *Evolution* 63 663–673. 10.1111/j.1558-5646.2008.00573.x 19220456

[B3] Allard P. M. Péresse T. Bisson J. Gindro K. Marcourt L. Pham V. C. (2016). Integration of molecular networking and in-silico MS/MS fragmentation for natural products dereplication. *Anal. Chem.* 88 3317–3323. 10.1021/acs.analchem.5b04804 26882108

[B4] Appendino G. (2016). “Ingenane diterpenoids,” in *Progress in the Chemistry of Organic Natural Products*, eds Kinghorn A. D. Falk H. Gibbons S. Kobayashi J. (Cham, CH: Springer International Publishing), 1–90. 10.1007/978-3-319-33172-0_1 27380406

[B5] Ayres M. P. Clausen T. P. MacLean S. F. Redman A. M. Reichardt P. B. (1997). Diversity of structure and antiherbivore activity in condensed tannins. *Ecology* 78 1696–1712. 10.1890/0012-9658(1997)078

[B6] Batra S. W. T. (1983). Establishment of Hyles Euphorbiae (L.) *(Lepidoptera: Sphingidae*) in the United States for control of the weedy spurges *Euphorbia esula* L. and *E. cyparissias* L. *J. N. Y. Entomol. Soc.* 91 304–311.

[B7] Becerra J. X. (1997). Insects on plants: macroevolutionary chemical trends in host use. *Science* 276 253–256. 10.1126/science.276.5310.253 9092474

[B8] Becerra J. X. (2007). The impact of herbivore–plant coevolution on plant community structure. *Proc. Natl. Acad. Sci. U.S.A.* 104 7483–7488. 10.1073/pnas.0608253104 17456606 PMC1855276

[B9] Bednarek P. Osbourn A. (2009). Plant-microbe interactions: chemical diversity in plant defense. *Science* 324 746–748. 10.1126/science.1171661 19423814

[B10] Berman B. (2012). New developments in the treatment of actinic keratosis: focus on ingenol mebutate gel. *Clin. Cosmet. Investig. Dermatol.* 20 111–122. 10.2147/CCID.S28905 22956883 PMC3430094

[B11] Birmingham A. Selfors L. M. Forster T. Wrobel D. Kennedy C. J. Shanks E. (2009). Statistical methods for analysis of high-throughput RNA interference screens. *Nat. Methods* 6 569–575. 10.1038/nmeth.1351 19644458 PMC2789971

[B12] Bouslimani A. Porto C. Rath C. M. Wang M. Guo Y. Gonzalez A. (2015). Molecular cartography of the human skin surface in 3D. *Proc. Natl. Acad. Sci. U.S.A.* 112 E2120–E2129. 10.1073/pnas.1424409112 25825778 PMC4418856

[B13] Böcker S. Dührkop K. (2016). Fragmentation trees reloaded. *J. Cheminform.* 8:5. 10.1186/s13321-016-0116-8 26839597 PMC4736045

[B14] Brenes-Arguedas T. Horton M. W. Coley P. D. Lokvam J. Waddell R. A. Meizoso-O’Meara B. E. (2006). Contrasting mechanisms of secondary metabolite accumulation during leaf development in two tropical tree species with different leaf expansion strategies. *Oecologia* 149 91–100. 10.1007/s00442-006-0423-2 16676208

[B15] Cignoni P. Callieri M. Corsini M. Dellepiane M. Ganovelli F. Ranzuglia G. (2008). “Meshlab: an open-source mesh processing tool,” in *Proceedings of the Eurographics Italian Chapter Conference*, eds Scarano V. Chiara R. D. Erra U. (Genova: The Eurographics Association).

[B16] Coley P. Aide T. M. (1991). “Comparison of herbivory and plant defenses in temperate and tropical broad-leaved forests,” in *Plant-Animal Interactions: Evolutionary Ecology in Tropical and Temperate Regions*, eds Price P. W. Lewinsohn T. Fernandes G. W. Benson W. W. (New York, NY: Wiley & Sons), 25–49.

[B17] Coley P. D. Barone J. A. (1996). Herbivory and plant defenses in tropical forests. *Annu. Rev. Ecol. Syst.* 27 305–335. 10.1146/annurev.ecolsys.27.1.305

[B18] da Silva O. L. M. Cordeiro I. (2016). Euphorbia sarcoceras, a new species of Euphorbia sect. Alectoroctonum from Brazil. *Syst. Bot.* 40 962–967. 10.3389/fpls.2018.00660 29868103 PMC5968112

[B19] da Silva R. R. Wang M. Nothias L. F. van der Hooft J. J. J. Caraballo-Rodríguez A. M. Fox E. (2018). Propagating annotations of molecular networks using in silico fragmentation. *PLoS Comput. Biol.* 14:e1006089. 10.1371/journal.pcbi.1006089 29668671 PMC5927460

[B20] Djoumbou Feunang Y. Eisner R. Knox C. Chepelev L. Hastings J. Owen G. (2016). ClassyFire: automated chemical classification with a comprehensive, computable taxonomy. *J. Cheminform.* 8:61. 27867422 10.1186/s13321-016-0174-y PMC5096306

[B21] Dorsey B. L. Haevermans T. Aubriot X. Morawetz J. J. Riina R. Steinmann V. W. (2013). Phylogenetics, morphological evolution, and classification of Euphorbia subgenus Euphorbia (Euphorbiaceae). *TAXON* 62 291–315. 10.12705/622.1

[B22] Dührkop K. Shen H. Meusel M. Rousu J. Böcker S. (2015). Searching molecular structure databases with tandem mass spectra using CSI: fingerid. *Proc. Natl. Acad. Sci. U.S.A.* 112 12580–12585. 10.1073/pnas.1509788112 26392543 PMC4611636

[B23] Dyer L. A. Philbin C. S. Ochsenrider K. M. Richards L. A. Massad T. J. Smilanich A. M. (2018). Modern approaches to study plant–insect interactions in chemical ecology. *Nat. Rev. Chem.* 2 50–64.

[B24] Ehrlich P. R. Raven P. H. (1964). Butterflies and plants: a study in coevolution. *Evolution* 18 586–608. 10.1111/j.1558-5646.1964.tb01674.x

[B25] Engelberth J. (2015). “23: Biotic interactions,” in *Plant Physiology and Development*, eds Taiz L. Zeiger E. Møller I. M. Murphy A. (Sunderland, MA: Sinauer Associates, Incorporated), 693–729.

[B26] Ernst M. Nothias L. F. van der Hooft J. J. J. Silva R. R. Saslis-Lagoudakis C. H. Grace O. M. (2018). Did a plant-herbivore arms race drive chemical diversity in Euphorbia? *bioRxiv*

[B27] Firn R. D. Jones C. G. (2003). Natural products - a simple model to explain chemical diversity. *Nat. Prod. Rep.* 20 382–391. 12964834 10.1039/b208815k

[B28] Floros D. J. Petras D. Kapono C. A. Melnik A. V. Ling T. J. Knight R. (2017). Mass spectrometry based molecular 3D-cartography of plant metabolites. *Front. Plant Sci.* 8:429. 10.3389/fpls.2017.00429 28405197 PMC5370242

[B29] Govaerts R. Dransfield J. Zona S. Hodel D. R. Henderson A. (2014). *World Checklist of Euphorbiaceae.* Sydney: Royal Botanic Gardens.

[B30] Horn J. W. van Ee B. W. Morawetz J. J. Riina R. Steinmann V. W. Berry P. E. (2012). Phylogenetics and the evolution of major structural characters in the giant genus Euphorbia L. *(Euphorbiaceae)*. *Mol. Phylogenet. Evol.* 63 305–326. 10.1016/j.ympev.2011.12.022 22273597

[B31] Horn J. W. Xi Z. Riina R. Peirson J. A. Yang Y. Dorsey B. L. (2014). Evolutionary bursts in Euphorbia (Euphorbiaceae) are linked with photosynthetic pathway. *Evolution* 68 3485–3504. 10.1111/evo.12534 25302554

[B32] Hua J. Liu Y. Xiao C. J. Jing S. X. Luo S. H. Li S. H. (2017). Chemical profile and defensive function of the latex of *Euphorbia peplus*. *Phytochemistry* 136 56–64. 10.1016/j.phytochem.2016.12.021 28062071

[B33] Huang C. S. Luo S. H. Li Y. L. Li C. H. Hua J. Liu Y. (2014). Antifeedant and antiviral diterpenoids from the fresh roots of *Euphorbia jolkinii*. *Nat. Prod. Bioprospect.* 4 91–100. 10.1007/s13659-014-0009-3 24859600 PMC4004854

[B34] Hundsdoerfer A. K. Kitching I. J. Wink M. (2005a). The phylogeny of the hyles euphorbiae complex (Lepidoptera: Sphingidae): molecular evidence from sequence data and ISSR-PCR fingerprints. *Org. Divers. Evol.* 5 173–198. 10.1016/j.ode.2004.11.012

[B35] Hundsdoerfer A. K. Tshibangu J. N. Wetterauer B. Wink M. (2005b). Sequestration of phorbol esters by aposematic larvae of *Hyles Euphorbiae* (Lepidoptera: Sphingidae): *Chemoecology* 15 261–267. 10.1007/s00049-005-0321-9

[B36] Hundsdoerfer A. K. Rubinoff D. Attieì M. Wink M. Kitching I. J. (2009). A revised molecular phylogeny of the globally distributed hawkmoth genus Hyles (Lepidoptera: Sphingidae), based on mitochondrial and nuclear DNA sequences. *Mol. Phylogenet. Evol.* 52 852–865. 10.1016/j.ympev.2009.05.023 19482093

[B37] Jian B. Zhang H. Liu J. (2018). Structural diversity and biological activities of diterpenoids derived from *Euphorbia fischeriana* Steud. *Molecules* 23:935. 10.3390/molecules23040935 29669996 PMC6017929

[B38] Jones C. G. Firn R. D. (1991). On the evolution of plant secondary chemical diversity. *Philos. Trans. R. Soc. Lond. B Biol. Sci.* 333 273–280. 10.1098/rstb.1991.0077

[B39] Kalliolias G. D. Ivashkiv L. B. (2016). TNF biology, pathogenic mechanisms and emerging therapeutic strategies. *Nat. Rev. Rheumatol.* 12 49–62. 10.1038/nrrheum.2015.169 26656660 PMC4809675

[B40] Krenzelok E. P. Jacobsen T. Aronis J. M. (1996). Poinsettia exposures have good outcomes just as we thought. *Am. J. Emerg. Med.* 14 671–674. 10.1016/s0735-6757(96)90086-8 8906768

[B41] Kursar T. A. Dexter K. G. Lokvam J. Pennington R. T. Richardson J. E. Weber M. G. (2009). The evolution of antiherbivore defenses and their contribution to species coexistence in the tropical tree genus Inga. *Proc. Natl. Acad. Sci. U.S.A.* 106 18073–18078. 10.1073/pnas.0904786106 19805183 PMC2775284

[B42] Loutit B. D. Louw G. N. Seely M. K. (1987). First approximation of food preferences and the chemical composition of the diet of the desert-dwelling black rhinoceros, *Diceros bicornis* L. *Madoqua* 15 35–54.

[B43] McKey D. (1979). “The distribution of secondary compounds within plants,” in *Herbivores: Their Interaction with Secondary Plant Metabolites*, eds Rosenthal G. A. Janzen D. H. (Cambridge, MA: Academic Press), 56–134.

[B44] Meerman J. C. (1993). Relationships within the Hyles Euphorbiae-complex: a numerical taxonomy approach (Lepidoptera: Sphingidae). *Entomol. Gaz.* 44 205–209.

[B45] Monaco C. Nanchahal J. Taylor P. Feldmann M. (2014). Anti-TNF therapy: past, present and future. *Int. Immunol.* 27 55–62. 10.1093/intimm/dxu102 25411043 PMC4279876

[B46] Nothias-Scaglia L. F. Schmitz-Afonso I. Renucci F. Roussi F. Touboul D. Costa J. (2015). Insights on profiling of phorbol, deoxyphorbol, ingenol and jatrophane diterpene esters by high performance liquid chromatography coupled to multiple stage mass spectrometry. *J. Chromatogr. A* 1422 128–139. 10.1016/j.chroma.2015.09.092 26522744

[B47] Orme D. Freckleton R. Thomas G. Petzoldt T. Fritz S. Isaac N. (2013). *caper: Comparative Analyses of Phylogenetics and Evolution in R. R package version 0.5.2*. Available at: http://CRAN.Rproject.org/package=caper

[B48] Peirson J. A. Bruyns P. V. Riina R. Morawetz J. J. Berry P. E. (2013). A molecular phylogeny and classification of the largely succulent and mainly African Euphorbia subg. *Athymalus* (Euphorbiaceae). *TAXON* 62 1178–1199. 10.12705/626.12

[B49] Pluskal T. Castillo S. Villar-Briones A. Orešič M. (2010). MZmine 2: modular framework for processing, visualizing, and analyzing mass spectrometry-based molecular profile data. *BMC Bioinformatics* 11:395. 10.1186/1471-2105-11-395 20650010 PMC2918584

[B50] Protsyuk I. Melnik A. V. Nothias L. F. Rappez L. Phapale P. Aksenov A. A. (2018). 3D molecular cartography using LC–MS facilitated by optimus and ’ili software. *Nat. Protoc.* 13 134–154. 10.1038/nprot.2017.122 29266099

[B51] Revell L. J. (2012). phytools: an R package for phylogenetic comparative biology (and other things). *Methods Ecol. Evol.* 3 217–223. 10.1111/j.2041-210x.2011.00169.x

[B52] Richards L. A. Dyer L. A. Forister M. L. Smilanich A. M. Dodson C. D. Leonard M. D. (2015). Phytochemical diversity drives plant–insect community diversity. *Proc. Natl. Acad. Sci. U.S.A.* 112 10973–10978. 10.1073/pnas.1504977112 26283384 PMC4568244

[B53] Riina R. Peirson J. A. Geltman D. V. Molero J. Frajman B. Pahlevani A. (2013). A worldwide molecular phylogeny and classification of the leafy spurges, Euphorbia subgenus Esula (Euphorbiaceae). *TAXON* 62 316–342. 10.12705/622.3

[B54] Röst H. L. Sachsenberg T. Aiche S. Bielow C. Weisser H. Aicheler F. (2016). OpenMS: a flexible open-source software platform for mass spectrometry data analysis. *Nat. Methods* 13 741–748. 10.1038/nmeth.3959 27575624

[B55] Sedger L. M. McDermott M. F. (2014). TNF and TNF-receptors: from mediators of cell death and inflammation to therapeutic giants - past, present and future. *Cytokine Growth Factor Rev.* 25 453–472. 10.1016/j.cytogfr.2014.07.016 25169849

[B56] Sedio B. E. (2017). Recent breakthroughs in metabolomics promise to reveal the cryptic chemical traits that mediate plant community composition, character evolution and lineage diversification. *New Phytol.* 214 952–958. 10.1111/nph.14438 28134431

[B57] Sedio B. E. Rojas Echeverri J. C. Boya P. C. A. Wright S. J. (2017). Sources of variation in foliar secondary chemistry in a tropical forest tree community. *Ecology* 98 616–623. 10.1002/ecy.1689 27984635

[B58] Shannon P. Markiel A. Ozier O. Baliga N. S. Wang J. T. Ramage D. (2003). Cytoscape: a software environment for integrated models of biomolecular interaction networks. *Genome Res.* 13 2498–2504. 10.1101/gr.1239303 14597658 PMC403769

[B59] Shi Q. W. Su X. H. Kiyota H. (2008). Chemical and pharmacological research of the plants in genus Euphorbia. *Chem. Rev.* 108 4295–4327. 18817355 10.1021/cr078350s

[B60] Smith S. E. Meldrum B. S. (1992). The protein kinase C activators, phorbol 12-myristate,13- acetate and phorbol 12,13-dibutyrate, are convulsant in the pico-nanomolar range in mice. *Eur. J. Pharmacol.* 213 133–135. 10.1016/0014-2999(92)90242-v 1499648

[B61] Sumner L. W. Amberg A. Barrett D. Beale M. H. Beger R. Daykin C. A. (2007). Proposed minimum reporting standards for chemical analysis chemical analysis working group (CAWG) metabolomics standards initiative (MSI). *Metabolomics* 3 211–221. 10.1007/s11306-007-0082-2 24039616 PMC3772505

[B62] Symonds M. R. E. Blomberg S. P. (2014). “A primer on phylogenetic generalised least squares,” in *Modern Phylogenetic Comparative Methods and Their Application in Evolutionary Biology: Concepts and Practice*, ed. Garamszegi L. Z. (Berlin: Springer), 105–130. 10.1007/978-3-662-43550-2_5

[B63] Thompson J. N. (2005a). Coevolution: the geographic mosaic of coevolutionary arms races. *Curr. Biol.* 15 R992–R994. 16360677 10.1016/j.cub.2005.11.046

[B64] Thompson J. N. (2005b). *The Geographic Mosaic of Coevolution, Interspecific Interactions.* Chicago, IL: University of Chicago Press.

[B65] Thompson J. N. (2008). *Coevolution, Cryptic Speciation, and the Persistence of Interactions.* Oakland, CA: University of California Press, 216–224.

[B66] Uto T. Qin G. W. Morinaga O. Shoyama Y. (2012). 17-Hydroxy-jolkinolide B, a diterpenoid from Euphorbia fischeriana, inhibits inflammatory mediators but activates heme oxygenase-1 expression in lipopolysaccharide-stimulated murine macrophages. *Int. Immunopharmacol.* 12 101–109. 10.1016/j.intimp.2011.10.020 22080918

[B67] van der Hooft J. J. J. Wandy J. Barrett M. P. Burgess K. E. V. Rogers S. (2016). Topic modeling for untargeted substructure exploration in metabolomics. *Proc. Natl. Acad. Sci. U.S.A.* 113 13738–13743. 10.1073/pnas.1608041113 27856765 PMC5137707

[B68] Vasas A. Hohmann J. (2014). Euphorbia diterpenes: isolation, structure, biological activity, and synthesis (2008-2012). *Chem. Rev.* 114 8579–8612. 10.1021/cr400541j 25036812

[B69] Vogg G. Achatz S. Kettrup A. Sandermann H. (1999). Fast, sensitive and selective liquid chromatographic-tandem mass spectrometric determination of tumor-promoting diterpene esters. *J. Chromatogr. A* 855 563–573. 10.1016/s0021-9673(99)00728-1 10519093

[B70] Wandy J. Zhu Y. van der Hooft J. J. J. Daly R. Barrett M. P. Rogers S. (2018). Ms2lda.org: web-based topic modelling for substructure discovery in mass spectrometry. *Bioinformatics* 34 317–318. 10.1093/bioinformatics/btx582 28968802 PMC5860206

[B71] Wang M. Carver J. J. Phelan V. V. Sanchez L. M. Garg N. Peng Y. (2016). Sharing and community curation of mass spectrometry data with global natural products social molecular networking. *Nat. Biotechnol.* 34 828–837. 10.1038/nbt.3597 27504778 PMC5321674

[B72] Watrous J. Roach P. Alexandrov T. Heath B. S. Yang J. Y. Kersten R. D. (2012). Mass spectral molecular networking of living microbial colonies. *Proc. Natl. Acad. Sci. U.S.A.* 109 E1743–E1752. 10.1073/pnas.1203689109 22586093 PMC3387089

[B73] Wink M. (2010). “Introduction: biochemistry, physiology and ecological functions of secondary metabolites,” in *Annual Plant Reviews: Biochemistry of Plant Secondary Metabolism*, Vol. 40 ed. Wink M. (Hoboken, NJ: Wiley-Blackwell), 1–19. 10.1002/9781444320503.ch1

[B74] Wubshet S. G. Schmidt J. S. Wiese S. Staerk D. (2013). High-resolution screening combined with HPLC-HRMS-SPE-NMR for identification of potential health-promoting constituents in sea aster and searocket – new nordic food ingredients. *J. Agric. Food Chem.* 61 8616–8623. 10.1021/jf402949y 23962163

[B75] Yang Y. Berry P. E. (2011). Phylogenetics of the *Chamaesyce clade* (Euphorbia, Euphorbiaceae): reticulate evolution and long-distance dispersal in a prominent C4 lineage. *Am. J. Bot.* 98 1486–1503. 10.3732/ajb.1000496 21875975

[B76] Yang Y. Riina R. Morawetz J. J. Haevermans T. Aubriot X. Berry P. E. (2012). Molecular phylogenetics and classification of Euphorbia subgenus Chamaesyce (Euphorbiaceae). *TAXON* 61 764–789. 10.1002/tax.614005

[B77] Zangerl A. R. Berenbaum M. R. (2003). Phenotype matching in wild parsnip and parsnip web- 696 worms: causes and consequences. *Evolution* 57 806–815. 10.1111/j.0014-3820.2003.tb00292.x 12778550

[B78] Zhang C. Y. Wu Y. L. Zhang P. Chen Z. Z. Li H. Chen L. X. (2019). Anti-inflammatory lathyrane diterpenoids from *Euphorbia lathyris*. *J. Nat. Prod.* 28 756–764. 10.1021/acs.jnatprod.8b00600 30817151

[B79] Zhang J. H. Chung T. D. Y. Oldenburg K. R. (1999). A simple statistical parameter for use in evaluation and validation of high throughput screening assays. *J. Biomol. Screen.* 4 67–73. 10.1177/108705719900400206 10838414

[B80] Zimmermann N. F. A. Ritz C. M. Hellwig F. H. (2010). Further support for the phylogenetic relationships within Euphorbia L. (Euphorbiaceae) from nrITS and trnL–trnF IGS sequence data. *Plant Syst. Evol.* 286 39–58. 10.1007/s00606-010-0272-7

