# Supplementary material for: Assessing Specialized Metabolite Diversity in the Cosmopolitan Plant Genus Euphorbia L
Source: Front Plant Sci. 2019 Jul 2;10:846. doi: 10.3389/fpls.2019.00846 (PMC6615404; doi:10.3389/fpls.2019.00846)
Supplement: Supplementary file 1 [file Data_Sheet_1.pdf]

Supplementary Material

Assessing specialized metabolite diversity in the cosmopolitan plant genus *Euphorbia* L.

Madeleine Ernst[1,2,3*†], Louis-Félix Nothias[2,3], Justin J. J. van der Hooft[4], Ricardo R. Silva[2,3] , C. Haris Saslis-Lagoudakis[1] , Olwen M. Grace[5] , Karen Martinez-Swatson[1] , Gustavo Hassemer[1,6] , Luís A. Funez[6] , Henrik T. Simonsen[7], Marnix H. Medema[4] , Dan Staerk[8] , Niclas Nilsson[9] , Paola Lovato[9] , Pieter C. Dorrestein[2,3,10*] and Nina Rønsted[1*]

The following Supplementary Material is available for this article:

Fig. S1 *Euphorbia* global mass spectral molecular network: visualization of *Euphorbia* subgeneric clades.

Fig. S2 *Euphorbia* global mass spectral molecular network with major chemical classes highlighted. *In silico* annotation resulted in putative identification of over 30% of the nodes within the mass spectral molecular network up to the level of chemical subclasses.

Fig. S3 3D molecular map of a *Euphorbia* diterpene in *Euphorbia horrida* with predominant occurrence in the roots.

Fig. S4 3D molecular map of a *Euphorbia* diterpene in *Euphorbia hirta* with predominant occurrence in the fruits/seeds.

Fig. S5 3D molecular map of a *Euphorbia* diterpene in *Euphorbia lathyris* with predominant occurrence in the young stems.

Fig. S6 3D molecular map of a milliamine C derivative in *Euphorbia milii* var. *hislopii* with predominant occurrence in the roots.

Fig. S7 3D molecular map of a milliamine C derivative in *Euphorbia milii* var. *hislopii* with predominant occurrence in the roots.

Fig. S8 3D molecular map of milliamine C derivative in *Euphorbia milii* var. *hislopii* with predominant occurrence in the roots.

Fig. S9 3D molecular map of milliamine C derivative in *Euphorbia milii* var. *hislopii* with predominant occurrence in the roots.

Fig. S10 Differential expression (MS1 intensities) of molecules annotated as *Euphorbia* diterpenoids in different plant parts of one representative species per *Euphorbia* subgeneric clade.

Fig. S11 Illustration of sampling locations for 3D mass spectral molecular cartography.

Fig. S12 Compound classes showing significant association to the number of TNF-α modulating fractions using phylogenetic generalized least squares regression analysis.

Table S1 Phylogenetic generalized least squares regression analysis (PGLS) between the number of TNF-α modulating fractions and the number of compounds per chemical subclass.

Methods S1 Supporting methods information for extract preparation for 3D mass spectral molecular cartography.

Methods S2 Supporting methods information for LC-MS/MS analysis.

Methods S3 Supporting methods information for LC-MS/MS analysis for 3D mass spectral molecular cartography.

Methods S4 Supporting methods information for mass spectral molecular networking.

Methods S5 Supporting methods information for mass spectral molecular networking for 3D mass spectral molecular cartography.

Methods S6 Supporting methods information for integration of *in silico* annotation, automated chemical classification and substructure recognition with mass spectral molecular networks for 3D mass spectral molecular cartography.

Methods S7 Supporting methods information for high-resolution microplate-based extract collection.

Methods S8 Supporting methods information for TNF-α inhibition assay using human peripheral blood mononuclear cells.

Notes S1 Extended description of mass spectral molecular network analysis.

URL S1 URL links of interactive 3D mass spectral molecular cartographical snapshots.

Data S1 List of *Euphorbia* species sampled (separate file).

Data S2 High-resolution TNF-α modulation profiles (separate file).

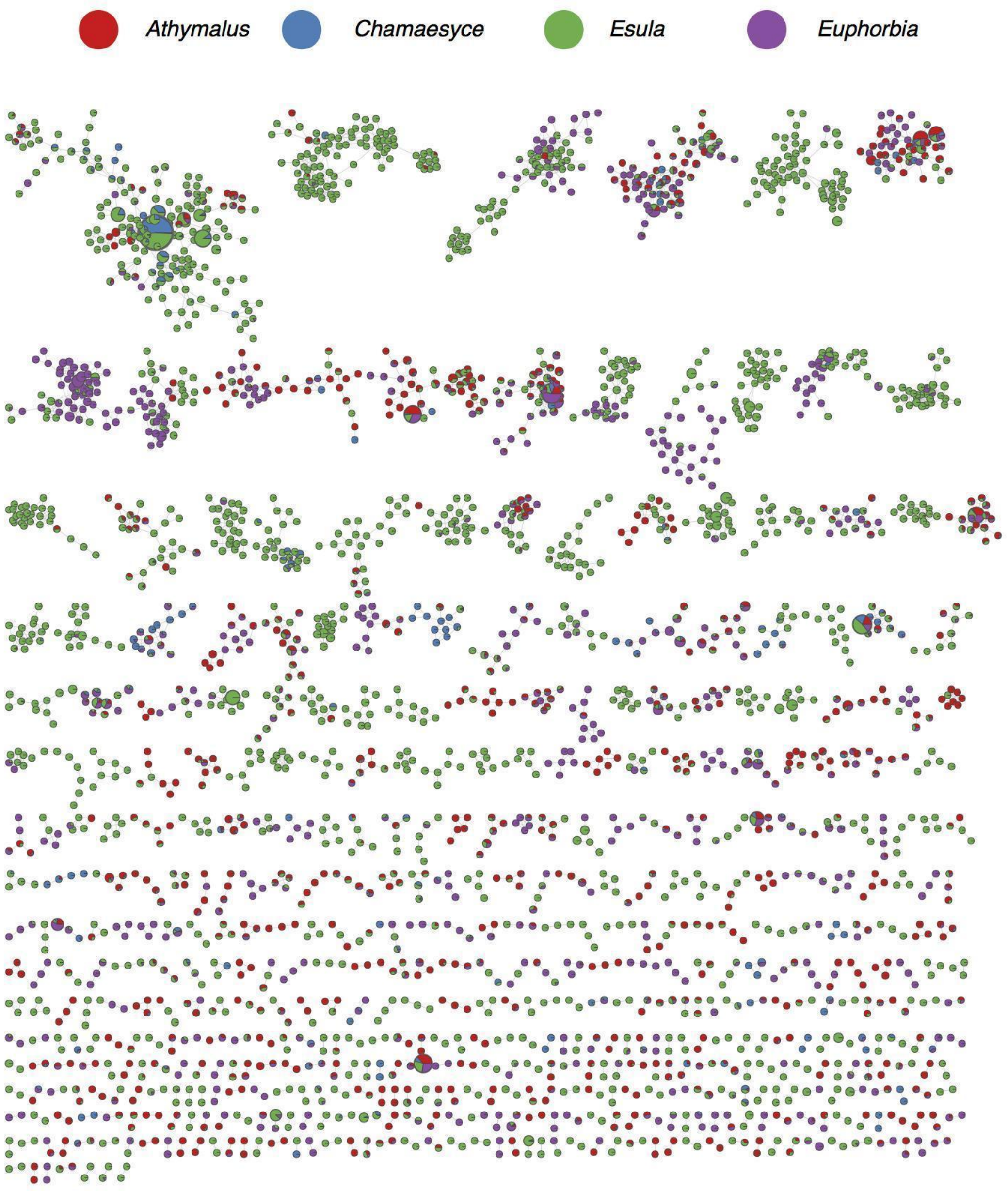

Fig. S1 *Euphorbia* global mass spectral molecular network: visualization of *Euphorbia* subgeneric clades. Pie charts represent total ion current (TIC) observed per subgeneric clade, node size represents TIC of all samples, the thickness of the lines connecting the nodes (edges) is a representative of the cosine score. Nodes showing no similarity to any other node are not shown.

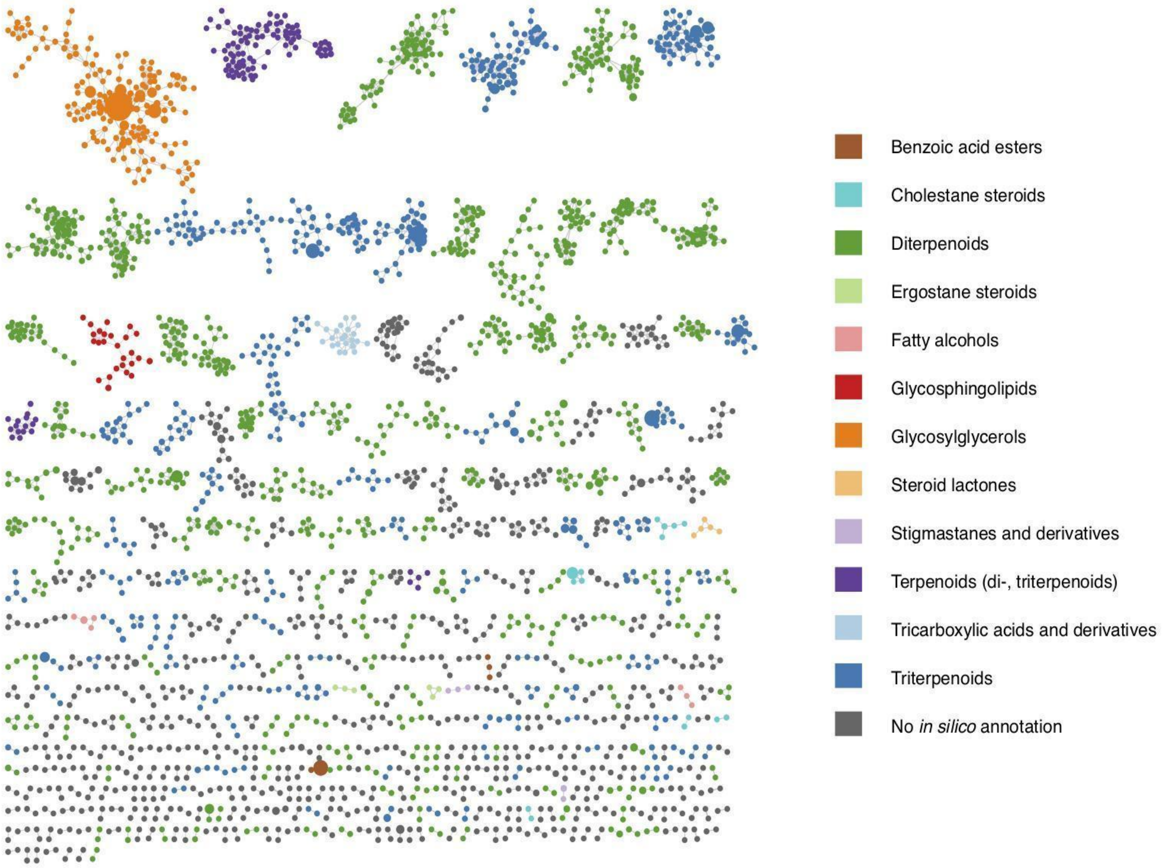

Fig. S2 *Euphorbia* global mass spectral molecular network with major chemical classes highlighted. *In silico* annotation resulted in putative identification of over 30% of the nodes within the mass spectral molecular network up to the level of chemical subclasses.

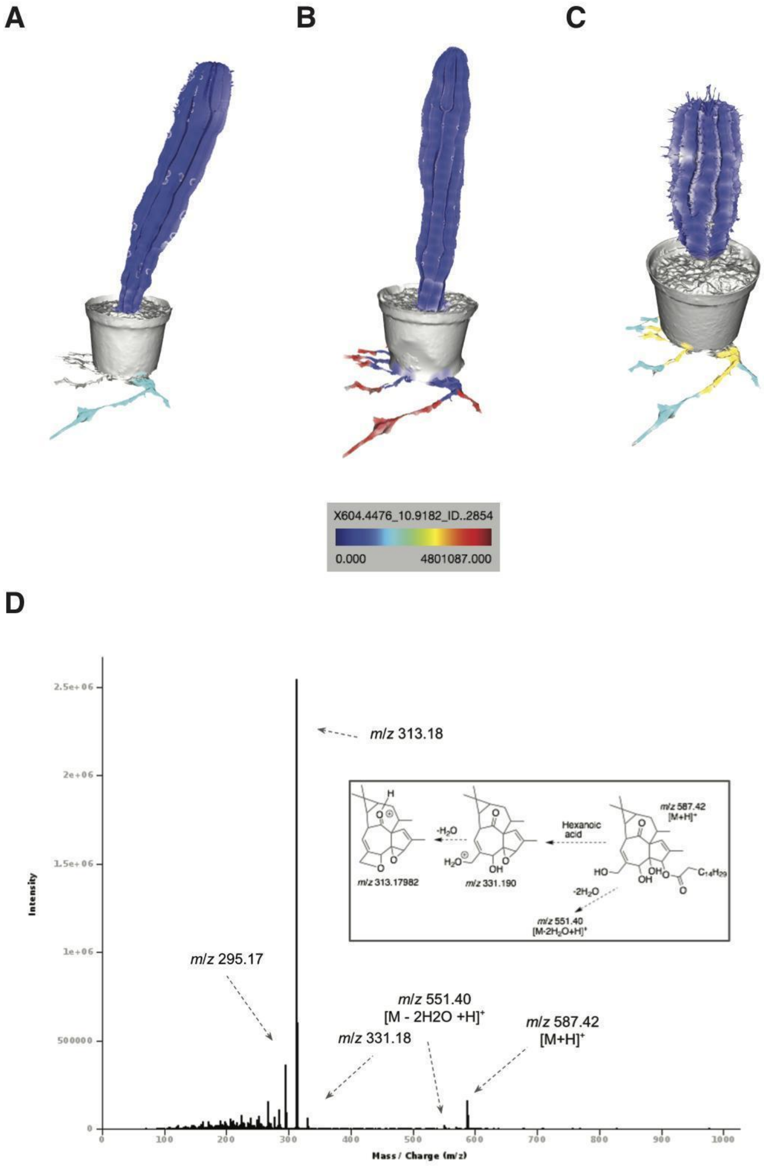

Fig. S3 3D molecular map of a *Euphorbia* diterpene in *Euphorbia horrida* with predominant occurrence in the roots. **A**. *Euphorbia horrida* Model 1. **B**. *Euphorbia horrida* Model 2. **C**. *Euphorbia horrida* Model 3. **D**. MS/MS spectrum of ingenol or deoxyphorbol ester with hexanoyl and putatively annotated features (http://gnps.ucsd.edu//ProteoSAFe/result.jsp?task=cbd1ea3bc17045188d2184c4289f3023&view=cluster_details&protein=2854). For interactive cartographical snapshots see URL S1, links 1-3.

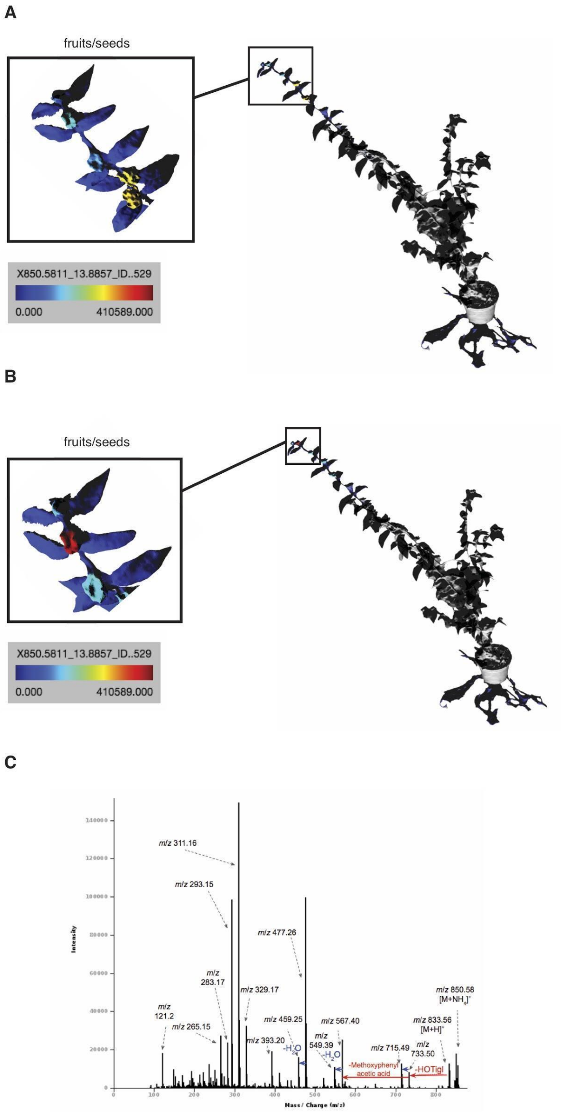

Fig. S4 3D molecular map of a *Euphorbia* diterpene in *Euphorbia hirta* with predominant occurrence in the fruits/seeds. **A**. *Euphorbia hirta* Model 1. **B**. *Euphorbia hirta* Model 2. **C**. MS/MS spectrum exhibiting diterpene spectral fingerprint of type A (DSF-A) with *m/z* 311, 293, 283 and partially annotated features (http://gnps.ucsd.edu//ProteoSAFe/result.jsp?task=cbd1ea3bc17045188d2184c4289f3023&view=cluster_details&protein=529). For interactive cartographical snapshots see URL S1, links 4-5.

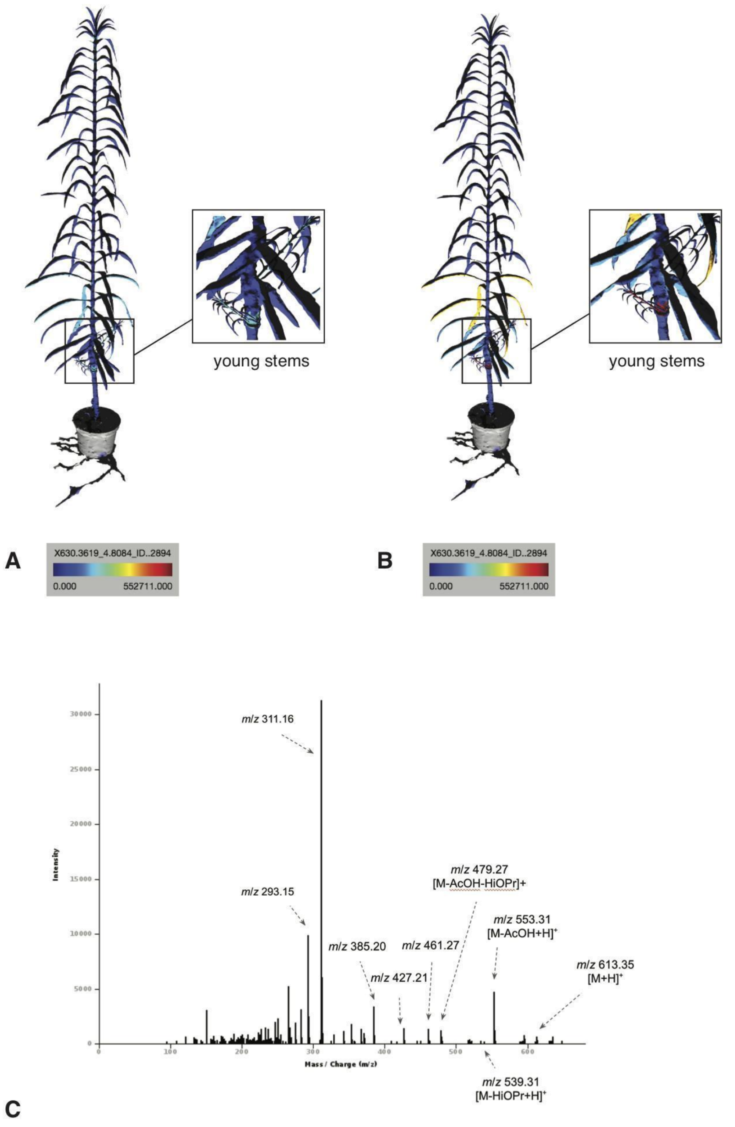

Fig. S5 3D molecular map of a *Euphorbia* diterpene in *Euphorbia lathyris* with predominant occurrence in the young stems. **A**. *Euphorbia lathyris* Model 1. **B**. *Euphorbia lathyris* Model 2. **C**. MS/MS spectrum exhibiting diterpene spectral fingerprint of type A (DSF-A) with *m/z* 311, 293, 283 (http://gnps.ucsd.edu//ProteoSAFe/result.jsp?task=cbd1ea3bc17045188d2184c4289f3023&view=cluster_details&protein=2894). For interactive cartographical snapshots see URL S1, links 6-7.

A

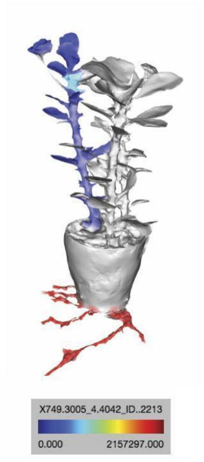

B

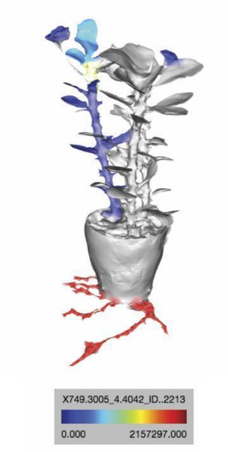

C

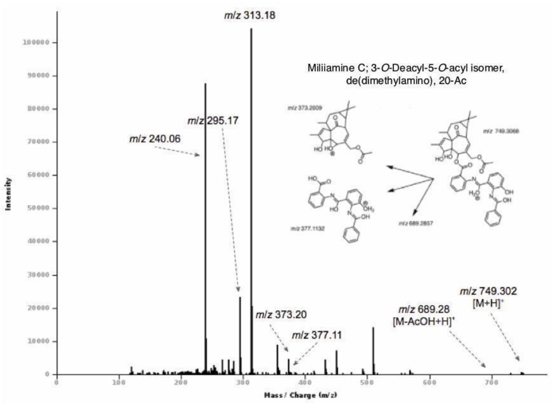

Fig. S6 3D molecular map of a milliamine C derivative in *Euphorbia milii* var. *hislopii* with predominant occurrence in the roots. **A**. *Euphorbia milii* var. *hislopii* Model 1. **B**. *Euphorbia milii* var. *hislopii* Model 2. Additionally to the roots, low occurrence of milliamine C derivative was also observed in the young stems and leaves. **C**. MS/MS spectrum annotated as milliamine C; 3-*O*-Deacyl-5-*O*-acyl isomer, de(dimethylamino), 20-Ac. The MS/MS spectrum can be accessed at: http://gnps.ucsd.edu//ProteoSAFe/result.jsp?task=cbd1ea3bc17045188d2184c4289f3023&view=cluster_details&protein=2213. For interactive cartographical snapshots see URL S1, links 8-9.

A

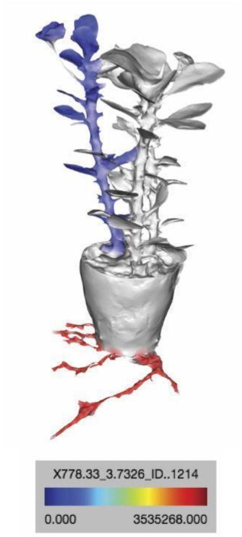

B

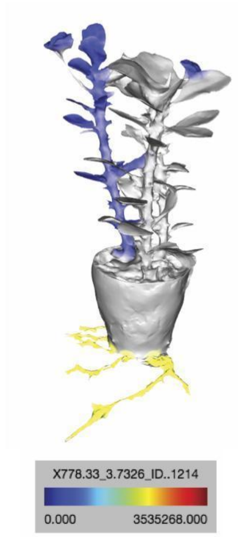

C

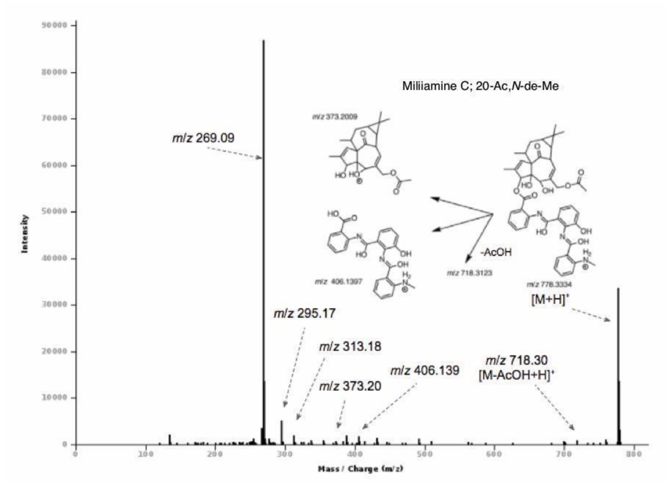

Fig. S7 3D molecular map of a milliamine C derivative in *Euphorbia milii* var. *hislopii* with predominant occurrence in the roots. A. *Euphorbia milii* var. hislopii Model 1. B. *Euphorbia milii* var. *hislopii* Model 2. C. MS/MS spectrum annotated as milliamine C; 20-Ac, *N*-de-Me. The MS/MS spectrum can be accessed at: http://gnps.ucsd.edu//ProteoSAFe/result.jsp?task=cbd1ea3bc17045188d2184c4289f3023&view=cluster_details&protein=1214. For interactive cartographical snapshots see URL S1, links 10-11.

A

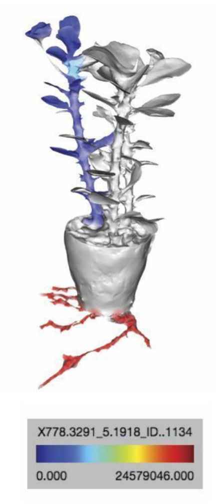

B

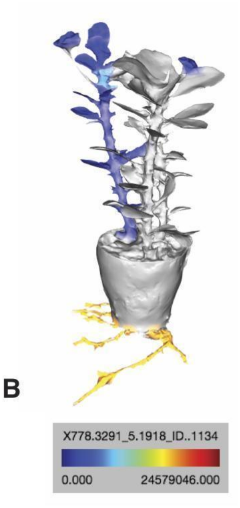

C

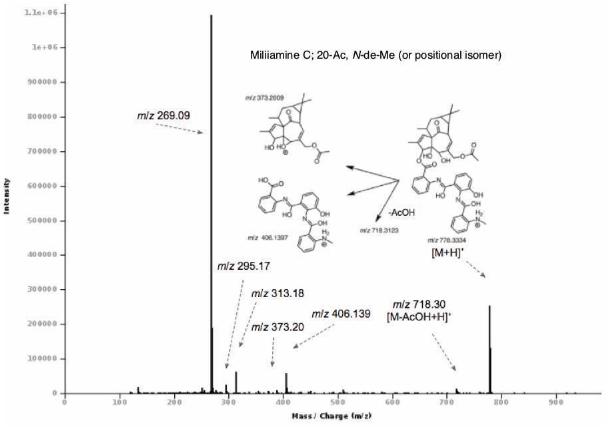

Fig. S8 3D molecular map of milliamine C derivative in *Euphorbia milii* var. *hislopii* with predominant occurrence in the roots. **A**. *Euphorbia milii* var. *hislopii* Model 1. **B**. *Euphorbia milii* var. *hislopii* Model 2. Additionally, to the roots, low occurrence of milliamine C was also observed in the young stems. **C**. MS/MS spectrum annotated as miliiamine C; 20-Ac, *N*-de-Me (or positional isomer). The MS/MS spectrum can be accessed at: http://gnps.ucsd.edu//ProteoSAFe/result.jsp?task=cbd1ea3bc17045188d2184c4289f3023&view=cluster_details&protein=1134. For interactive cartographical snapshots see URL S1, links 12-13.

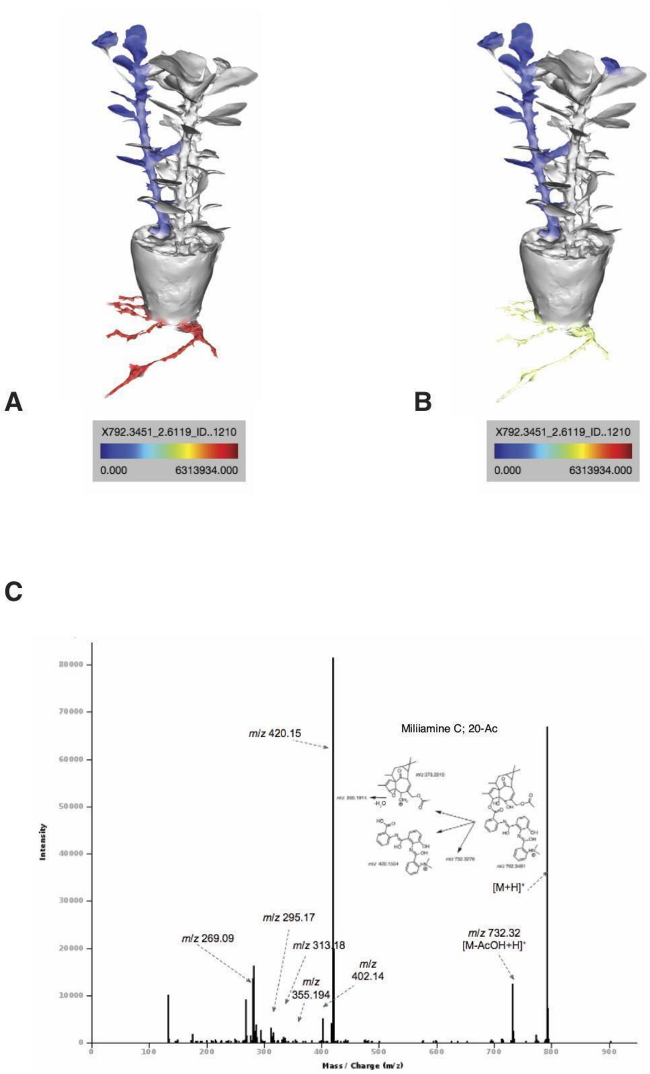

Fig. S9 3D molecular map of milliamine C derivative in *Euphorbia milii* var. *hislopii* with predominant occurrence in the roots. **A**. *Euphorbia milii* var. *hislopii* Model 1. **B**. *Euphorbia milii* var. *hislopii* Model 2. **C**. MS/MS spectrum annotated as milliamine C; 20-Ac. The MS/MS spectrum can be accessed: (http://gnps.ucsd.edu//ProteoSAFe/result.jsp?task=cbd1ea3bc17045188d2184c4289f3023&view=cluster_details&protein=1210). For interactive cartographical snapshots see URL S1, links 14-15.

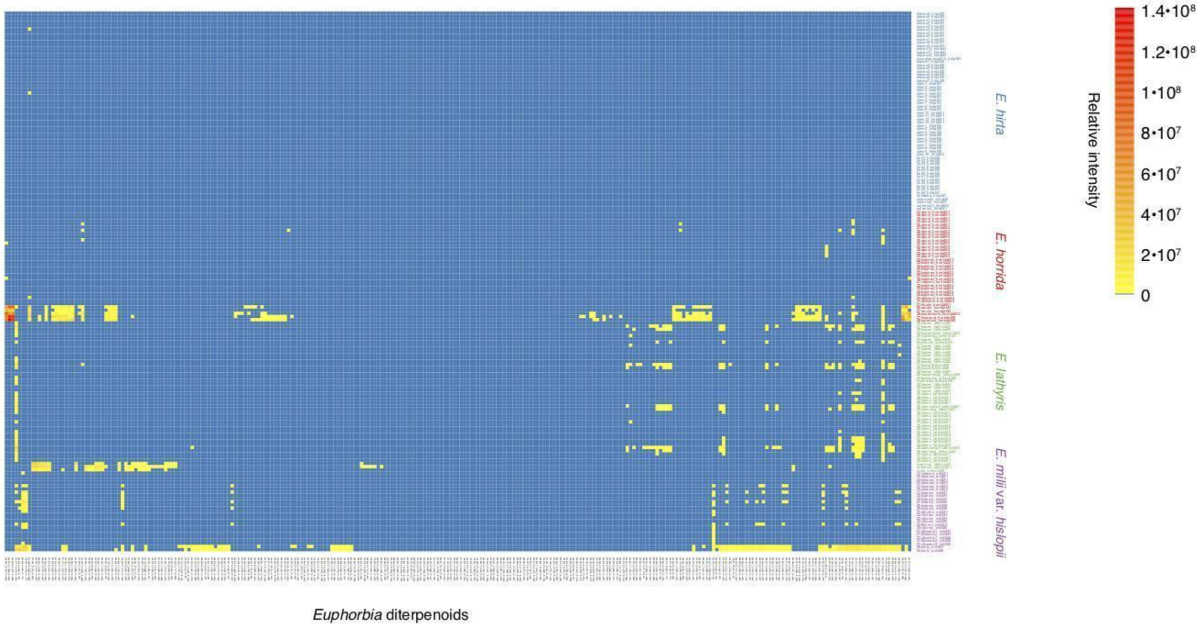

Fig. S10 Differential expression (MS1 intensities) of molecules annotated as *Euphorbia* diterpenoids in different plant parts of one representative species per *Euphorbia* subgeneric clade. *Euphorbia* diterpenoid production is reduced throughout the whole plant in *Euphorbia hirta*, a representative of the American radiation within subgenus *Chamaesyce* (blue). In *Euphorbia horrida* (subgenus *Athymalus*, red) and *Euphorbia milii* var. *hislopii* (subgenus *Euphorbia*, purple) *Euphorbia* diterpenoids were predominantly found in the roots (Fig. S3 and S6-S9) and in *Euphorbia lathyris* (Subgenus *Esula*, green) they were abundant in several plant parts, among others in the young leaves and stems (Fig. S5).

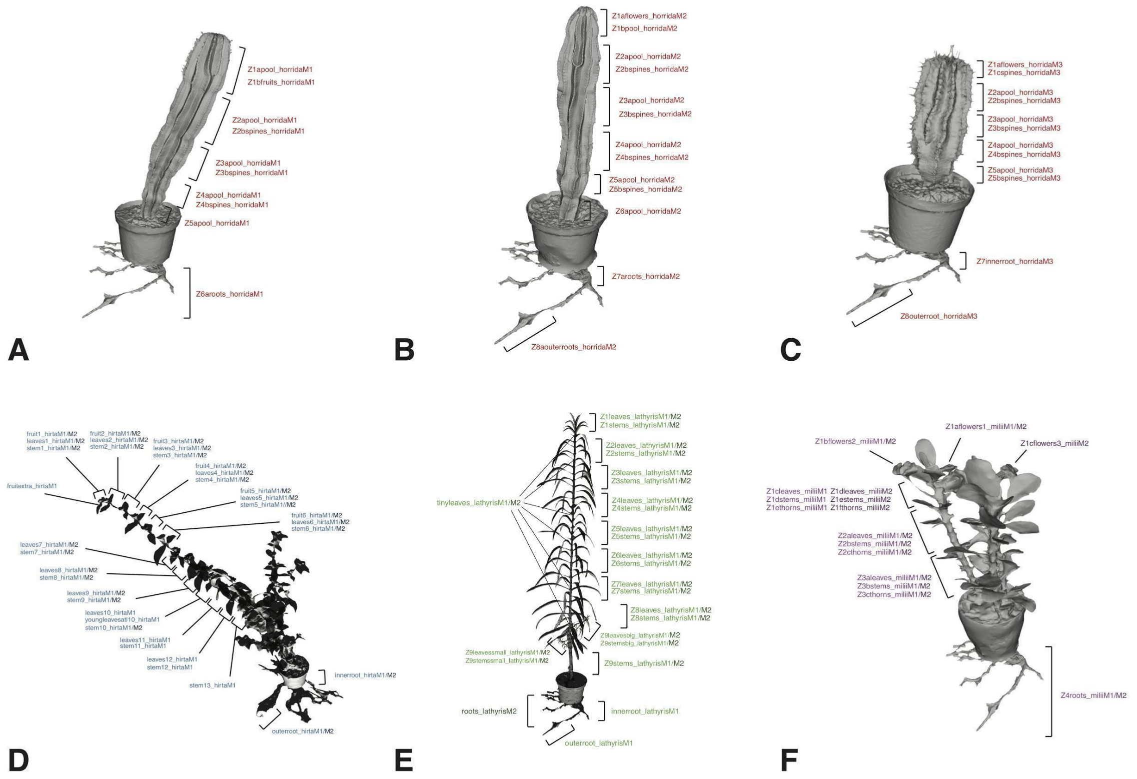

Fig. S11 Illustration of sampling locations for 3D mass spectral molecular cartography. Individual plant parts from one to three specimens of one representative species of each subgeneric clade of *Euphorbia* were sampled. **A**.- **C**. *E. horrida*, subgenus *Athymalus*, 3 specimens. LC-MS/MS data were mapped on three 3D Models of three individual specimens. **D.** *E. hirta*, *Chamaesyce*, 2 specimens. LC-MS/MS data were mapped on one representative 3D Model of one specimen; **E**. *E. lathyris*, *Esula*, 2 specimens. LC-MS/MS data were mapped on one representative 3D Model of one specimen. **F**. *E. milii* var. *hislopii*, *Euphorbia*, 2 specimens. LC-MS/MS data were mapped on one representative 3D Model of one specimen.

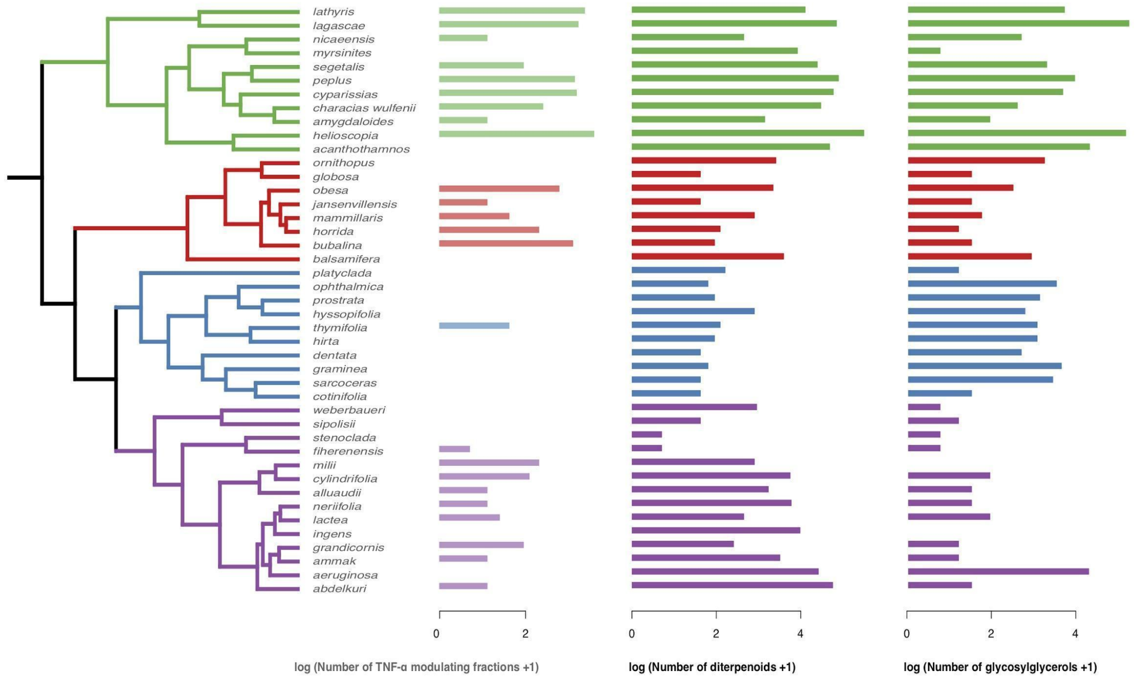

Fig. S12 Compound classes showing significant association to the number of TNF-α modulating fractions using phylogenetic generalized least squares regression analysis. *Euphorbia* phylogenetic tree (50% majority rule consensus tree from Bayesian analysis of 11587 bps of DNA markers spanning all three plant genomes) and number of TNF-*α* modulating fractions versus number of putatively annotated diterpenoids and glycosylglycerols per species analyzed.

Table S1 Phylogenetic generalized least squares regression analysis (PGLS) between the number of TNF-α modulating fractions and the number of compounds per chemical subclass.

**Chemical subclass**	**p-value**	**Adjusted R-squared**	
Benzoic acids and derivatives	0.79	-0.02	
Cholestane steroids	0.54	-0.01	
**Diterpenoids**	**0.02**	**0.11**	*
Glycosphingolipids	0.95	-0.02	
**Glycosylglycerols**	**0.02**	**0.10**	*
Stigmastanes and derivatives	0.16	0.02	
Triterpenoids	0.63	-0.02	
Regular diterpenoids	0.06	0.06	
***Euphorbia* diterpenoids**	**0.02**	**0.10**	*

Methods S1 Supporting methods information for extract preparation for 3D mass spectral molecular cartography.

The frozen plant material was disrupted in plastic tubes (Qiagen, RB 2 mL) in 1.3 ml 50/50 vol/vol methanol (Fisher Scientific, HPLC Grade)/acetonitrile (Fisher Scientific, Optima LC/MS) with stainless steel beads (VWR International, 5 mm) using a tissue lyser (Qiagen, TissueLyser II) at 25 Hz during 10 min. The samples were then extracted under sonication (Fisher Scientific) at 40°C during 15 min and centrifuged (Eppendorf Centrifuge 5418) for 10 min at 11,000 r.p.m. The extract supernatant was transferred to new plastic tubes and lyophilized to dryness (Labcono, Acid Resistant CentriVap Concentrator).

Methods S2 Supporting methods information for LC-MS/MS analysis.

Each sample was analyzed separately two times with two different MS-methods for MS acquisition. For the first MS-method, 5 µL were injected, auto-MS/MS was turned off, whereas for the second MS-method, 20 µL were injected and auto-MS/MS was activated. For both MS-methods, MS spectra were acquired in positive ion mode over a mass range of 75-1,000 *m/z*. An external calibration with sodium formate was performed before data acquisition and hexakis(1H,1H,3H-tetrafluoropropoxy)phosphazene (Synquest Laboratories) *m/z* 922.009798 was used as a lock mass internal calibrant during data acquisition. The following instrument settings were used for data acquisition: end plate Offset 500 V, capillary voltage of 5,000 V, nebulizer gas (nitrogen) pressure of 2.0 bar, ion source temperature of 200°C, dry gas flow of 9 l/min, source temperature and spectra acquisition rate of 4 Hz for MS1 and MS2. Tune parameters were set as

follows: Funnel RF1 200 Vpp, ion energy 3.0 V, Hexapole RF 80 Vpp, Prepulse storage 7 µs. Minutes 0-0.7 were sent to waste. For the MS-method with auto-MS/MS turned on, the three most intense ions per MS1 scan were selected and subjected to collision-induced dissociation if absolute intensity reached 12205 counts. The following fragmentation and isolation lists were used (values are *m/z*, isolation width and collision energy, respectively): 100, 2, 10; 250, 2, 15; 300, 2, 20; 400, 2, 20; 500, 2, 20; 600, 2, 30; 700, 2, 30; 800, 2, 30; 1000, 2, 40. In addition, the advanced stepping function was used with time, collision radiofrequency (RF), transfer time stepping (µs) and collision energy set to: 0, 800, 85, 75; 400, 65, 100, 100; 50, 400, 65, 100; 75, 150, 45, 150. The MS/MS active exclusion parameter was set to 1 and released after 0.25 min, and reseted if ion intensity was three times higher. The injected samples were chromatographically separated using an Agilent 1290 Infinity Binary LC System (Agilent Technologies) controlled by Hystar software (Bruker Daltonics), using a 100 x 2.1 mm Kinetex 1.7 µM, C18, 100 Å chromatography column (Phenomenex), 40°C column temperature, 0.5 ml/min flow rate, mobile phase A 99.9\% water (Fisher Scientific, Optima LC/MS)/0.1% formic acid (Fisher Scientific, Optima LC/MS)/10 mM ammonium formate (Fluka, LC-MS Ultra), mobile phase B 99.9% acetonitrile (Fisher Scientific, Optima LC/MS)/0.1% formic acid (Fisher Scientific, Optima LC/MS)/10 mM ammonium formate (Fluka, LC-MS Ultra), with the following gradient: 0-0.5 min 55% B, 0.5-15 min 100% B, 15-18.5 min 100% B, 18.5-20 min 100% B. Blanks were injected between each analyzed sample, and the column was equilibrated prior to any injection with the following gradient 20-20.2 min 55% B, 20.2-20.4 min 100% B, 20.4-23 min 55% B, 23-25 min 55%. Blank injections - 20 µL methanol:acetonitrile (3:7) used for extraction were used as negative controls. A representative extract was used as a quality control (QC) sample, and this QC sample was analyzed every twelve samples to monitor retention time shift and intensity shift.

Methods S3 Supporting methods information for LC-MS/MS analysis for 3D mass spectral molecular cartography.

Dried extracts were dissolved in 50/50 vol/vol methanol (Fisher Scientific, HPLC Grade)/acetonitrile (Fisher Scientific, Optima LC/MS) to a concentration of 1 mg/ml, 100 μL of each extract were transferred to a 96-well plate (Falcon, 96-well plates, 0.34 ml, polypropylene), sealed with Zone-Free Sealing Film (Excel Scientific) and centrifuged for 30 min. at 2000 r.p.m. at 4°C. 20 μL of each extract were injected into the LC-MS/MS equipment. MS analysis was performed on a micrOTOF-Q II (Bruker Daltonics) mass spectrometer with an electrospray ionization (ESI) source, controlled by OTOF control and Hystar. MS spectra were acquired in positive ion mode over a mass range of 75-1,000 *m/z*. An external calibration with sodium formate was performed before data acquisition and hexakis(1H,1H,3H-tetrafluoropropoxy)phosphazene (Synquest Laboratories) *m/z* 922.009798 was used as a lock mass internal calibrant during data acquisition. The following instrument settings were used for data acquisition: capillary voltage of 4,500 V, nebulizer gas (nitrogen) pressure of 2 bar, ion source temperature of 200°C, dry gas flow of 9 l/min, source temperature and spectra acquisition rate of 3 Hz for MS1 and MS2. Minutes 0-0.5 and 20-25 were sent to waste. Minutes 0.5-20 were recorded with auto MS/MS turned on. The three most intense ions per MS1 scan were selected and subjected to collision-induced dissociation according to the following fragmentation and isolation list (values are m/z, isolation width and collision energy, respectively): 100, 2, 10; 250, 3, 15; 300, 4, 15; 400, 4, 15; 500, 4, 15; 600, 5, 20; 700, 5, 20; 800, 5, 20; 1000, 7, 35; 100, 0, 5. In addition, the advanced stepping function was used with time, collision radiofrequency (RF), transfer time stepping (μs) and collision energy set to:

0, 550, 125, 75; 25, 400, 90, 100; 50, 250, 65, 125; 75, 150, 50, 150. The MS/MS active exclusion parameter was set to 1 and released after 0.25 min. The injected samples were chromatographically separated using an Agilent 1290 Infinity Binary LC System (Agilent Technologies) controlled by Hystar software (Bruker Daltonics), using a 50 x 2.1 mm Kinetex 1.7 µM, C18, 100 Å chromatography column (Phenomenex), 40°C column temperature, 0.5 ml min-1 flow rate, mobile phase A 99.9% water (Fisher Scientific, Optima LC/MS)/0.1% formic acid (Fisher Scientific, Optima LC/MS)/10 mM ammonium formate (Fluka, LC-MS Ultra), mobile phase B 99.9% acetonitrile (Fisher Scientific, Optima LC/MS)/0.1% formic acid (Fisher Scientific, Optima LC/MS)/10 mM ammonium formate (Fluka, LC-MS Ultra), with the following gradient: 0-0.5 min 55% B, 0.5-15 min 100% B, 15-18.5 min 100% B, 18.5-20 min 100% B, 20-20.2 min 55% B, 20.2-20.4 min 100% B, 20.4-23 min 55% B, 23-25 min 55% B. Blank injections - 20 µL methanol:acetonitrile (1:1) used for extraction - were used as controls. A representative extract was used as a quality control (QC) sample, and this QC sample was analyzed every twelve samples to monitor retention time shift and intensity shift.

Methods S4 Supporting methods information for mass spectral molecular networking.

Each sample was analyzed with two different acquisition methods, and the two LC-MS/MS data were merged using MassFuser (https://github.com/alexandrovteam/MassFuser), a dedicated software that combines the MS1 data from one run, with the MS2 data from the other run. Two complementary acquisition methods and different injection volumes were employed as described in the LC-MS/MS analysis section to acquire paired runs. This approach enabled acquisition of high-quality MS1 features with the first method (with a high frequency of MS1 scans per

chromatographic peak), and MS2 scans for a high number of MS1 features with the second method (including low intensity features). The corresponding paired data were combined with MassFuser. Merging failed for 6 out of 43 extracts, due to insufficient matching peaks in the low concentrated extracts *(E. acanthothamnos*, *E. aeruginosa*, *E. helioscopia*, *E. lagascae*, *E. ornithopus* and *E. balsamifera*). For these 6 extracts we used only the highly concentrated extracts for further analysis.

Methods S5 Supporting methods information for mass spectral molecular networking for 3D mass spectral molecular cartography.

LC-MS/MS data of the extracts for the 3D mass spectral molecular cartography were converted to mzML data, and lock mass correction was performed using Compass Data Analysis (Bruker Daltonics). The mzML files were then preprocessed using MZmine 2.3 (Pluskal *et al.,* 2010) with parameters set to: Peak detection/Mass detection/Mass detector, Centroid, MS1 noise level 6.0E3, MS2 noise level 100, Chromatogram builder/MS1 level, Min time span (min) 0.01, Min height 2.0E4, *m/z* tolerance 0.02 *m/z* or 20 ppm, Chromatogram deconvolution/Algorithm, Baseline cut-off, *m/z* range for MS2 scan pairing (Da) 0.03, RT range for MS2 scan pairing (min) 0.5, Min peak height, 2.0E4, Peak duration range (min) 0.01-4, Baseline level 6.0E3. Isotopic peak grouper, *m/z* tolerance 0.02 *m/z* or 20 ppm, Retention time tolerance 0.4 (min), Maximum charge 2, Representative isotopes, Most intense, Feature alignment, *m/z* tolerance 0.03 *m/z* or 30 ppm, Weight for *m/z* 75, Retention time tolerance 0.5, Weight for RT 25, Gap filling, Intensity tolerance 10%, *m/z* tolerance 0.02 *m/z* or 20 ppm, Retention time tolerance 0.4 (min), RT correction Off. Subsequently, the .mgf output file was uploaded to the Global Natural Products Social Molecular

Networking web server (http://gnps.ucsd.edu) and submitted to network analysis using the following settings: Precursor Ion Mass Tolerance 0.02 Da, Fragment Ion Mass Tolerance 0.02 Da, Min Pairs Cos 0.5, Min Matched Fragment Ions 6, Network TopK 10, Minimum Cluster Size 1, Maximum Connected Component Size 200, Run MS Cluster off. The molecular networks were visualized using Cytoscape version 3.4.0 (Shannon *et al.*, 2003). The data are publicly accessible at http://gnps.ucsd.edu under the MassIVE accession no. MSV000081083 and network exploration options and views are available and networking parameters described at: https://gnps.ucsd.edu/ProteoSAFe/status.jsp?task=cbd1ea3bc17045188d2184c4289f3023

Methods S6 Supporting methods information for integration of *in silico* annotation, automated chemical classification and substructure recognition with mass spectral molecular networks for 3D mass spectral molecular cartography.

Data from 3D mass spectral molecular cartography were submitted to Network Annotation Propagation (NAP) (da Silva *et al.*, 2018). Both [M+NH4]+ and [M+H]+ adducts were searched with *m/z* tolerance set to 20 ppm and parameters described at: https://proteomics2.ucsd.edu/ProteoSAFe/status.jsp?task=6d58b49a645d408e9471f82d1b308f6 and https://proteomics2.ucsd.edu/ProteoSAFe/status.jsp?task=425dd24f42d54b4ba58aba69f2f1dfac. The same custom database of molecular structures as described for the pooled extracts was matched against our samples' mass spectral data. Subsequently, *in silico* structure matches from NAP were submitted to automated chemical classification using ClassyFire (http://classyfire.wishartlab.com/) (Djoumbou Feunang *et al.*, 2016) and consensus classifications

at each hierarchical level of the chemical taxonomy per mass spectral molecular subnetwork were calculated. Consensus classifications and molecular structures were then visualized on the molecular networks using Cytoscape version 3.4.0 (Shannon *et al.*, 2003).

Methods S7 Supporting methods information for high-resolution microplate-based extract collection.

HPLC analyses of the extracts were performed on an Agilent 1200 series chromatograph consisting of a G1311A quaternary pump, a G1322A degasser, a G1316A thermostated column compartment, a G1315C photodiode-array detector, a G1367C high-performance autosampler, and a G1364C fraction collector (Santa Clara, CA). Separations were performed with a Phenomenex Luna C18(2) column (150 x 4.6 mm i.d., 3 μm particle size, 100 Å pore size) maintained at 40°C. HPLC solvent A consisted of water-acetonitrile 95:5 with 0.1% formic acid added, and solvent B consisted of acetonitrile-water 95:5 with 0.1% formic acid added. The dried extracts were resuspended in acetonitrile and 0.8 mg of extract was injected and separated by use of the following gradient elution profile at 0.5 ml/min: 0 min, 0% B; 22.5 min, 100% B; 40 min, 100% B; 41 min, 0% B; 48 min, 0% B. The chromatograms were monitored at 254, 226, 235, 280 and 360 nm. The chromatographic system was controlled by the Agilent ChemStation revision B.03.02 software. Fractionation was performed from 10 to 45 min in 144 wells. Microplates were evaporated to dryness under reduced pressure in an SPD121P Savant SpeedVac.

Methods S8 Supporting methods information for TNF-α inhibition assay using human

peripheral blood mononuclear cells.

Human peripheral blood mononuclear cells (hPBMCs) were isolated from human buffy coats of healthy donors using Lymphoprep (Medinor) according to manufacturer's instructions and frozen at $5 \cdot 10^7$ cells/ml in (RPMI cell medium with 20% Fetal Calf Serum (FCS), (Gibco) and 5% DMSO). The fractionated dry extracts were resuspended in 30 μL DMSO, and two times 70 nl was transferred to 384-well assay plates using the Echo (Labcyte) liquid handling equipment. Anti-Biotin MACSiBead Particles loaded with biotinylated antibodies against human CD3 and CD28 were used to mimic antigen-presenting cells and activate resting T cells from PBMCs. The beads were loaded with biotinylated antibodies according to the manufacturer's instructions (human T Cell Activation/Expansion Kit, Milteny Biotec). The cells were thawed, and beads in a 1:1 bead-to-cell ratio were added before seeding 70 μl of $73.5 \cdot 10^3$ cells per well to the assay plates. The assay plates were incubated 24 h overnight after which 5 μl of supernatant were transferred to a 384-well detection plate to measure the level of TNF-α by using the AlphaLisa kit for human TNF-α (Perkin-Elmer) according to the manufacturer's protocol. Percentage effect on TNF-α inhibition was calculated by using DMSO and T cell activated PBMCs as 0% effect for no activity, i.e. maximal TNF-α release and 10 μM terfenadine and T cell activated PBMCs as 100% effect for full inhibition, i.e. no TNF-α detected. 10 μM dexamethasone was added to each assay plate as a control for a potent compound reducing TNF-α levels without exhibiting effects on cell viability. To evaluate possible cytotoxicity, we also measured inhibition of cell viability. The inhibition of cell viability was determined by measuring fluorescence after adding PrestoBlue (LifeScience) ready-to-use reagent to each well and incubating 24 h in the dark at room temperature according to the manufacturer's protocol. In order to evaluate the quality and

resolution of each assay plate we calculated the Z'-factor (Zhang *et al.,* 1999) for TNF-α modulation as well as cell viability (Data S1). The Z'-factor is defined as

$$Z'=1-3(\sigma_{c+}+\sigma_{c-})/|\mu_{c+}-\mu_{c-}|$$

where σ_{c+} indicates the standard deviation of 100% effect, σ_{c-} indicates the standard deviation of 0% effect, μ_{c+} indicates the mean value of 100% effect and μ_{c-} indicates the mean value of 0% effect. Assay performance was considered very good for Z' > 0.5 (Birmingham *et al.,* 2009).

Ingenol mebutate is a *Euphorbia* diterpenoid with known anti-herbivore activity (Hua *et al.,* 2017). Therefore, we subjected ingenol mebutate to the same assay setup as the plant extracts. Ingenol mebutate (Data S2, Fig. 1) did not show a dose-dependent inhibition or increase of TNF-α release. Nevertheless, significant modulation of TNF-α was observed, demonstrating that ingenol mebutate like diterpenoids can be pinpointed with the chosen assay setup. We fit a symmetric log-logistic model to our raw data of ingenol mebutate as well as the crude plant extracts (Data S2), none of the extracts however showed a significant fit to the model. Calculated values for model fits are therefore not shown. TNF-α modulatory activity was assessed for each fraction, with two replicate experiments per fraction. TNF-α modulatory activity and cell viability were plotted against chromatographic retention time to give a high-resolution biochromatogram (Data S2), with individual measurements represented as dots and mean values as bars. Fractions with mean values for TNF-α modulation of > 35% and < -35% respectively and cell viability not exceeding (-)30% were considered as significantly modulating the release of TNF-α without having a significant effect on cell viability. Cell viability baseline was observed to be lower than -30% in some of the experiments. In these cases, we also considered

fractions as significantly modulating TNF-α if their percentage of modulation was 1.5 times higher than of the cell viability. Fractions considered significantly modulating TNF-α are highlighted in Data S2, Figs. 2-45.

Notes S1 Extended description of mass spectral molecular network analysis.

The mass spectral molecular network of the pooled extracts consisted of 5,652 nodes, organized in 512 independent molecular families, comprising a total of 2,739 nodes (two or more connected components of a graph) (Fig. S1). Automated chemical classification of the *in silico* annotated structures through ClassyFire (Djoumbou Feunang *et al.*, 2016) resulted in putatively identified compound classes for over 1,800 nodes in the network, corresponding to a level 3 metabolite identification according to the Metabolomics Standard Initiative's reporting standards (Sumner *et al.*, 2007). We manually validated *in silico* structure annotation at the subclass level and the direct parent level for the diterpenoids, which led to reclassification in some cases. Detailed description of the interpretation of the *in silico* annotated compound classes is found in Cytoscape_SummaryTable.csv under the column headers 'Subclass_Interpretation' and 'Diterpenoid_Subclass_Interpretation' available at https://github.com/DorresteinLaboratory/supplementary-GlobalEuphorbiaStudy.

The mass spectral molecular network of the extracts used for 3D mass spectral molecular cartography consisted of 5,335 nodes, organized in 435 independent molecular families, comprising a total of 3,318 nodes (two or more connected components of a graph). Automated chemical classification of the *in silico* annotated structures through ClassyFire (Djoumbou Feunang *et al.*, 2016) resulted in putatively identified compound classes for over 2,000 nodes in

the network, corresponding to a level 3 metabolite identification according to the Metabolomics Standard Initiative's reporting standards (Djoumbou Feunang *et al.*, 2016). We manually validated *in silico* structure annotation for the *Euphorbia* diterpenoids, which led to reclassification in some cases. Manually validated *Euphorbia* diterpenoids can be found in Cytoscape_SummaryTable_3DMolecularCartography.csv under the column header 'Diterpenoid_Subclass_Interpretation' available at https://github.com/DorresteinLaboratory/supplementary-GlobalEuphorbiaStudy.

URL S1 URL links of interactive 3D mass spectral molecular cartographical snapshots.

The URL links listed below can be opened in a web browser. Each of the links stores a snapshot of the view shown in Fig.S3-S9. Clicking on the URL opens the 'ili application and loads the data settings. The visualizations are interactive: one can rotate the model, change the parameters of the visualization or visualize different molecular features based on *m/z* and retention time values (Protsyuk *et al.,* 2018).

1. *Euphorbia horrida*, Model 1, Fig. S3

https://ili.embl.de/?ftp://massive.ucsd.edu/MSV000081081/updates/2017-05-15_mernst_9ac10437/peak/EHorrida_Model1_WithRoots.stl;ftp://massive.ucsd.edu/MSV000081081/updates/2018-03-21_mernst_c88e7520/peak/X604.4476_10.9182_ID..2854_Horrida1.json;ftp://massive.ucsd.edu/MSV000081081/updates/2018-03-21_mernst_c88e7520/peak/EHorrida_Model1_features_MZmine.csv

2. *Euphorbia horrida*, Model 2, Fig. S3

https://ili.embl.de/?ftp://massive.ucsd.edu/MSV000081081/updates/2017-05-15_mernst_9ac10437/peak/EHorrida_20160915_Model2_Clone_withroots.stl;ftp://massive.ucsd.edu/MSV000081081/updates/2018-03-21_mernst_c88e7520/peak/X604.4476_10.9182_ID..2854_Horrida2.json;ftp://massive.ucsd.edu/MSV000081081/updates/2018-03-21_mernst_c88e7520/peak/EHorrida_Model2_features_MZmine.csv

3. *Euphorbia horrida*, Model 3, Fig. S3

https://ili.embl.de/?ftp://massive.ucsd.edu/MSV000081081/updates/2017-05-15_mernst_9ac10437/peak/EHorrida_20160915_Model3_withroots.stl;ftp://massive.ucsd.edu/MSV000081081/updates/2018-03-21_mernst_c88e7520/peak/X604.4476_10.9182_ID..2854_Horrida3.json;ftp://massive.ucsd.edu/MSV000081081/updates/2018-03-21_mernst_c88e7520/peak/EHorrida_Model3_features_MZmine.csv

4. *Euphorbia hirta*, Model 1, Fig. S4

https://ili.embl.de/?ftp://massive.ucsd.edu/MSV000081081/updates/2017-05-15_mernst_9ac10437/peak/EHirta_20160908_Model2.stl;ftp://massive.ucsd.edu/MSV000081081/updates/2018-03-21_mernst_c88e7520/peak/X850.5811_13.8857_ID..529_Hirta1.json;ftp://massive.ucsd.edu/MSV000081081/updates/2018-03-21_mernst_c88e7520/peak/EHirta_Model1_features_MZmine.csv

5. *Euphorbia hirta*, Model 2, Fig. S4

https://ili.embl.de/?ftp://massive.ucsd.edu/MSV000081081/updates/2017-05-15_mernst_9ac10437/peak/EHirta_20160908_Model2.stl;ftp://massive.ucsd.edu/MSV000081081/updates/2018-03-21_mernst_c88e7520/peak/X850.5811_13.8857_ID..529_Hirta2.json;ftp://massive.ucsd.edu/MSV000081081/updates/2018-03-21_mernst_c88e7520/peak/EHirta_Model2_features_MZmine.csv

6. *Euphorbia lathyris*, Model 1, Fig. S5

https://ili.embl.de/?ftp://massive.ucsd.edu/MSV000081081/peak/ELathyris_Final.stl;ftp://massive.ucsd.edu/MSV000081081/updates/2018-03-21_mernst_c88e7520/peak/X630.3619_4.8084_ID..2894_Lathyris1.json;ftp://massive.ucsd.edu/MSV000081081/updates/2018-03-21_mernst_c88e7520/peak/ELathyris_Model1_features_MZmine.csv

7. *Euphorbia lathyris*, Model 2, Fig. S5

https://ili.embl.de/?ftp://massive.ucsd.edu/MSV000081081/peak/ELathyris_Final.stl;ftp://massive.ucsd.edu/MSV000081081/updates/2018-03-21_mernst_c88e7520/peak/X630.3619_4.8084_ID..2894_Lathyris2.json;ftp://massive.ucsd.edu/MSV000081081/updates/2018-03-21_mernst_c88e7520/peak/ELathyris_Model2_features_MZmine.csv

8. *Euphorbia milii*, Model 1, milliamine C, Fig. S6

https://ili.embl.de/?ftp://massive.ucsd.edu/MSV000081081/updates/2017-05-15_mernst_9ac10437/peak/EMilii_withRoots.stl;ftp://massive.ucsd.edu/MSV000081081/updates/2018-03-21_mernst_c88e7520/peak/X749.3005_4.4042_ID..2213_Milii1.json;ftp://massive.ucsd.edu/MSV000081081/updates/2018-03-21_mernst_c88e7520/peak/EMilii_Model1_features_MZmine.csv

9. *Euphorbia milii*, Model 2, milliamine C, Fig. S6

https://ili.embl.de/?ftp://massive.ucsd.edu/MSV000081081/updates/2017-05-15_mernst_9ac10437/peak/EMilii_withRoots.stl;ftp://massive.ucsd.edu/MSV000081081/updates/2018-03-21_mernst_c88e7520/peak/X749.3005_4.4042_ID..2213_Milii2.json;ftp://massive.ucsd.edu/MSV000081081/updates/2018-03-21_mernst_c88e7520/peak/EMilii_Model2_features_MZmine.csv

10. *Euphorbia milii*, Model 1, milliamine C, Fig. S7

https://ili.embl.de/?ftp://massive.ucsd.edu/MSV000081081/updates/2017-05-15_mernst_9ac10437/peak/EMilii_withRoots.stl;ftp://massive.ucsd.edu/MSV000081081/updates/2018-04-30_mernst_ec3691ad/peak/X778.33_3.7326_ID..1214_Milii1.json;ftp://massive.ucsd.edu/MSV000081081/updates/2018-03-21_mernst_c88e7520/peak/EMilii_Model1_features_MZmine.csv

11. *Euphorbia milii*, Model 2, milliamine C, Fig. S7

https://ili.embl.de/?ftp://massive.ucsd.edu/MSV000081081/updates/2017-05-15_mernst_9ac10437/peak/EMilii_withRoots.stl;ftp://massive.ucsd.edu/MSV000081081/updates/2018-04-30_mernst_ec3691ad/peak/X778.33_3.7326_ID..1214_Milii2.json;ftp://massive.ucsd.edu/MSV000081081/updates/2018-03-21_mernst_c88e7520/peak/EMilii_Model2_features_MZmine.csv

12. *Euphorbia milii*, Model 1, milliamine C, Fig. S8

https://ili.embl.de/?ftp://massive.ucsd.edu/MSV000081081/updates/2017-05-15_mernst_9ac10437/peak/EMilii_withRoots.stl;ftp://massive.ucsd.edu/MSV000081081/updates/2018-04-30_mernst_ec3691ad/peak/X778.3291_5.1918_ID..1134_Milii1.json;ftp://massive.ucsd.edu/MSV000081081/updates/2018-03-21_mernst_c88e7520/peak/EMilii_Model1_features_MZmine.csv

13. *Euphorbia milii*, Model 2, milliamine C, Fig. S8

https://ili.embl.de/?ftp://massive.ucsd.edu/MSV000081081/updates/2017-05-15_mernst_9ac10437/peak/EMilii_withRoots.stl;ftp://massive.ucsd.edu/MSV000081081/updates/2018-04-30_mernst_ec3691ad/peak/X778.3291_5.1918_ID..1134_Milii2.json;ftp://massive.ucsd.edu/MSV000081081/updates/2018-03-21_mernst_c88e7520/peak/EMilii_Model2_features_MZmine.csv

14. *Euphorbia milii*, Model 1, milliamine C, Fig. S9

https://ili.embl.de/?ftp://massive.ucsd.edu/MSV000081081/updates/2017-05-15_mernst_9ac10437/peak/EMilii_withRoots.stl;ftp://massive.ucsd.edu/MSV000081081/updates/2018-04-30_mernst_ec3691ad/peak/X792.3451_2.6119_ID..1210_Milii1.json;ftp://massive.ucsd.edu/MSV000081081/updates/2018-03-21_mernst_c88e7520/peak/EMilii_Model1_features_MZmine.csv

15. *Euphorbia milii*, Model 2, milliamine C, Fig. S9

https://ili.embl.de/?ftp://massive.ucsd.edu/MSV000081081/updates/2017-05-15_mernst_9ac10437/peak/EMilii_withRoots.stl;ftp://massive.ucsd.edu/MSV000081081/updates/2018-04-30_mernst_ec3691ad/peak/X792.3451_2.6119_ID..1210_Milii2.json;ftp://massive.ucsd.edu/MSV000081081/updates/2018-03-21_mernst_c88e7520/peak/EMilii_Model2_features_MZmine.csv

Data S1. List of *Euphorbia* species sampled (separate file).

Data S2. High-resolution TNF-α modulation profiles (separate file).
